# Probing the binding stability of organic UV filters to human SLC transporters using AlphaFold and molecular dynamics simulations

**DOI:** 10.1038/s41598-026-56714-z

**Published:** 2026-06-05

**Authors:** Ruikai Xing, Zhongquan Jiang, Tianyi Wei, Jiale Chen, Lijun Zhang, Shuangqing Hu, Zhemin Shen, Tao Yuan

**Affiliations:** 1 Key Laboratory of Environmental Health Impact Assessment of Emerging Contaminants, Ministry of Ecology and Environment, School of Environmental Science and Engineering, Shanghai Jiao Tong University, Shanghai, 200240 China; 2 East China Sea Fisheries Research Institute, Chinese Academy of Fishery Sciences, Shanghai, 200090 China; 3 School of Big Data and Artificial Intelligence, Chizhou University, Chizhou, 247000 China; 4 Division of Public Health Service and Safety Assessment, Shanghai Municipal Center for Disease Control and Prevention, Shanghai, 201107 China; 5 Shanghai Academy of Environmental Sciences, Shanghai, 200233 China

**Keywords:** Solute carrier transporters, Molecular dynamics simulation, Molecular docking, Organic ultraviolet filters, Biochemistry, Biophysics, Chemical biology, Chemistry, Computational biology and bioinformatics, Drug discovery

## Abstract

The ubiquitous presence of organic ultraviolet filters (UVFs) in environmental and human, driven by their heavy usage in personal care formulations, has raised significant concern. Despite this, the transmembrane transport mechanisms of UVFs—key to understanding cutaneous absorption and organ-specific toxicity—remain poorly defined. To bridge this gap, this study coupled molecular docking with molecular dynamics (MD) simulations to systematically screen the interactions between various UVFs and solute carrier (SLC) transporters. Through the analysis of 23,970 complex pairs, we identified distinct binding behaviors. Specifically, Tris-biphenyl triazine (TBPT) and Diethylhexyl butamido triazone (DBT) formed highly stable complexes with SLC22A31 and SLC29A4, respectively, exhibiting strong binding affinities (− 10.8 and − 9.8 kcal/mol). Over 200 ns simulations, these complexes maintained ligand Root Mean Square Deviation (RMSD) values below 5 Å. Crucially, per-residue interaction profiling revealed that this persistent stability is governed by high-occupancy (> 80%) hydrophobic anchors, which form rigid “hydrophobic traps” around the lipophilic UVFs. Conversely, the Pentyl 4-(dimethylamino) benzoate (APABA) and TMEM163 complex demonstrated weak interaction (− 3.9 kcal/mol) with significant structural instability, indicated by progressive ligand escape and RMSD values exceeding 15 Å. These findings elucidate how specific UVF-SLC interactions, driven by local hydrophobic complementarity, may facilitate localized toxicological effects, offering a novel computational framework for assessing the transmembrane health risks associated with organic UVFs exposure.

**Supplementary Information:**

The online version contains supplementary material available at 10.1038/s41598-026-56714-z.

## Introduction

The emerging contaminants of organic ultraviolet filters (UVFs) have been increasingly concerned in recent years. As essential components of sunscreen products, they are widely incorporated into various personal care products such as shampoos and sunscreens due to their efficient ultraviolet absorption capacity, which help to reduce the risks of photoaging and skin cancer^1^. However, growing evidence indicates that the extensive use of organic UVFs also poses non-negligible environmental and health risks. Reports have shown that the mass fractions of organic UVFs can reach the µg/kg level in dust, soil, and sediments, and their concentrations in surface waters can be as high as mg/L level, highlighting their strong environmental persistence and bioavailability^2–8^. In our previous studies, we revealed that organic UVFs exposure induces the production of inflammatory cytokines in human macrophages, identified multiple organic UVFs in indoor environments, with detection rates surpassing 92% in various indoor air samples^9^. We further found that the total concentration of four commonly used organic UVFs, namely 2-hydroxy-4-methoxybenzophenone (BP-3), 4-methylbenzylidene camphor (4-MBC), homosalate (HMS), and octocrylene (OC), in indoor dust ranged from 66.6 to 56,123.0 ng/g^10^. Moreover, these emerging contaminants may eventually be transmitted into the human body through multiple pathways, leading to potential toxic effects^11^. Some other experimental studies have also confirmed that organic UVFs can penetrate the skin and have been detected in both urine and blood^12,13^. Within organisms, organic UVFs might interact with multiple proteins, disrupting their normal functions^14^. Experimental evidence indicates that they can bind to digestive enzymes such as trypsin, inhibit their enzymatic activity, and thereby perturb normal protein metabolic processes^1^. Notably, many widely used UVFs (such as benzophenones and camphor derivatives) are recognized as endocrine-disrupting chemicals (EDCs). They exhibit estrogenic, anti-androgenic, or thyroid-disrupting activities, raising significant concerns regarding reproductive and developmental toxicity^15^. Meanwhile, nephrotoxicity was also observed in zebrafish toxicological experiments^16^. As mentioned above, UVFs exposure can affect a wide variety of target organs or tissues. Transmembrane behavior of UVFs is one of the important mechanisms to understand the skin exposure risks and assess the toxic potentials of target organs or tissues. However, the information of UVFs transmembrane behaviors is still scarce to date, although one of our previous studies confirmed the inhibitory effects of UVFs on multixenobiotic resistance (MXR) transporters, which belong to ATP-binding cassette (ABC) family proteins^17^.

On the other hand, as a core component of the human membrane transport network, the solute carrier (SLC) family comprises 470 transporters across 76 families^18^. Members of this superfamily are broadly responsible for the facilitated and coupled transmembrane transport of endogenous substrates and exogenous small molecules, thereby serving as critical targets for pharmacokinetic regulation and toxicological evaluation^19–21^. In contrast, ABC transporters perform primary active efflux via ATP hydrolysis, while channel proteins mainly provide passive permeation pathways; collectively, these three protein families determine the cellular uptake and clearance landscape^22^. When potentially small toxic molecules bind to SLC proteins, they can via mechanisms such as competitive inhibition and inappropriate influx, disrupt cellular metabolism and transmembrane homeostasis. Since SLC proteins are broadly expressed in human tissues and play critical roles in transmembrane solute transport, their interactions with UVFs could provoke toxic effects in different organs or tissues^23–25^. Experimental studies have confirmed that organic UVFs can penetrate the epidermis in vitro, and molecular docking analyses have demonstrated their ability to bind to certain SLC proteins with measurable binding affinity^14^. Such interactions may disrupt the normal substrate transport functions of SLC proteins, thereby altering cellular metabolic homeostasis and potentially inducing chronic toxicity or organ dysfunction^26–28^. Given these concerns, systematic characterization of UVF interactions with human SLC proteins is urgently needed.

Unfortunately, due to current technical difficulties, the purification and structural characterization of cellular membrane proteins remain challenging. Moreover, membrane protein structures account for only 1–2% of entries in the Protein Data Bank (PDB)^29^. Thus, it is not feasible to systematically investigate the interaction behaviors between SLC proteins and UVFs under in vivo and in vitro experimental models. However, computational science and simulation tools have presented dramatic development in the past decade. They provide essential opportunities for systematically studying SLC proteins and UVFs interactions. To overcome these experimental restrictions and ensure structural reliability, the present study employed predicted protein structures obtained from the AlphaFold Protein Structure Database for computational simulations^30^. AlphaFold has demonstrated near-experimental accuracy in multiple international assessments, such as the Critical Assessment of Protein Structure Prediction (CASP), and is particularly suitable for analyzing functional domain conformations and subsequent molecular simulations when high-resolution structural data are unavailable^31^. Meanwhile, compared with traditional in vivo and in vitro approaches, computational toxicology has emerged as a powerful tool for studying the biological effects of chemicals due to its high throughput, low cost, and strong reproducibility^32^. Especially in the context of complex interactions between multiple organic UVFs and diverse SLC subfamilies, computational approaches based on molecular docking and molecular dynamics (MD) simulations can accurately predict binding sites, binding affinity, and conformational changes between ligands and target proteins at the atomic scale^33^. These methods not only greatly improve research efficiency by enabling the systematic screening of large sets of candidate molecules and target proteins, but also circumvent common challenges in experimental studies of membrane proteins, including difficulties in expression, poor stability, and structural inaccessibility^34^. Furthermore, by constructing dynamic models of binding conformations and interaction energies, computational simulations can help identify key binding residues, predict risks of protein functional perturbation, and provide directed guidance for experimental validation^35^.

Therefore, the aim of this work is to generate standardized docking and dynamics data on UVF–SLC interactions, providing an innovative reference framework for toxicological studies and regulatory risk assessment. Based on the AlphaFold protein platform and molecular computation tools, this study employed molecular docking to screen and analyze the binding affinity and conformational characteristics of representative organic UVFs with selected SLC family proteins. Furthermore, MD simulations were performed to investigate and validate the stability of these interactions, identify key amino acid residues, and explore potential conformational changes. By constructing a molecular-level model of toxicological interactions, this work improves the understanding of complex physiological disturbances induced by these emerging contaminants in the human body. The findings are expected to provide a scientific basis for elucidating toxicological mechanisms and conducting risk assessment, as well as theoretical support for future environmental management and UVFs product safety evaluation.

## Materials and methods

### Acquisition of molecular files

The UVF molecules were obtained from two sources: (1) approved organic UVFs listed in the regulations of China, the European Union, and the United States^36–38^; (2) naturally occurring organic UVFs reported in the literature^39^. The three-dimensional structures of the UVF molecules were downloaded in SDF format from the PubChem database. For molecules lacking available 3D structural files, energy minimization was performed using Chem3D to obtain their lowest-energy conformations. This geometry optimization was performed using the MM2 force field. The structural minimization was iterated until the default convergence criterion was met, defined as a root-mean-square (RMS) gradient of less than 0.010 kcal/(mol·Å), thereby yielding the lowest-energy conformations for subsequent docking.

The list of all human SLC family members was collected from the HUGO Gene Nomenclature Committee (HGNC) database^40^. To ensure the highest biological relevance for our computational workflow, we systematically curated the protein structural dataset by prioritizing experimentally resolved structures. Through a comprehensive search of the Protein Data Bank (PDB)^41^, we retrieved available high-resolution experimental structures for 10 of these human SLC transporters. For the remaining 460 SLC transporters that currently lack experimental structures, their corresponding predicted models were downloaded from the AlphaFold Protein Structure Database^30^ to enable a superfamily-wide screening.

In total, 51 organic UVFs and 470 human SLC proteins were identified in this study. Detailed information of the 51 UVFs is provided in Table S1, while the complete list of the 470 SLC proteins is documented in Table S2.

### Molecular docking and molecular dynamics simulations

To investigate the binding characteristics between organic UVFs and SLC proteins, this study employed a computational approach combining molecular docking and MD simulations.

In this study, molecular docking was performed using AutoDock Vina 1.2.0^42^ to evaluate the binding affinity and conformational stability between macromolecular receptors and small-molecule ligands. To improve docking efficiency and meet the requirements of high-throughput screening across the structurally diverse human SLC superfamily, batch automation scripts were developed to carry out combinatorial docking tasks. Specifically, an unbiased blind docking strategy was implemented. The workflow included the following steps: first, all receptors and ligands were preprocessed using AutoDockTools, including the addition of polar hydrogens, calculation of Gasteiger charges, and conversion into PDBQT format. To ensure reproducibility across all 470 targets, the AutoDock Vina grid box parameters were dynamically assigned via our scripts. For each protein, the grid box center (coordinates) was set to the geometric center of the macromolecule. The grid size (dimensions in X, Y, and Z axes) was calculated to encompass the entire protein structure, with an additional buffer of 15 Å applied in all directions to provide sufficient space for ligand sampling. The docking search was executed with an exhaustiveness parameter of 8. For each docking run, AutoDock Vina was set to generate a maximum of 9 binding poses (num_modes = 9) within an energy range of 3 kcal/mol. All docking results were subsequently processed through an automated pipeline to extract the single pose with the lowest binding affinity score (the most stable conformation). This optimal pose inherently defined the selected binding pocket for subsequent statistical and visual analyses. Due to the lack of experimentally resolved co-crystal structures for these AlphaFold-predicted SLC models, traditional redocking validation was unfeasible. Therefore, blind docking was employed strictly as an initial hypothesis generator. The rigorous validation of these predicted binding poses, as well as the elimination of false-positive artifacts, was entirely entrusted to the subsequent 200 ns MD simulations.

MD simulations were performed using the AMBER 25 software^43^. To accurately capture the native-like conformational dynamics of the transporters, the systems were evaluated in an explicit lipid bilayer environment. Because the AlphaFold-predicted structures lacked predefined membrane coordinates, their spatial orientations relative to the hydrophobic core of the lipid bilayer were rigorously calculated using the PPM (Positioning of Proteins in Membranes) server. Following proper spatial alignment, the oriented protein-ligand complexes were embedded into a homogeneous POPC lipid bilayer using the Membrane Builder module of CHARMM-GUI^44,45^. The protein components were parameterized with the Amber ff14SB force field, while the small-molecule ligands were parameterized using the General Amber Force Field (GAFF) via the Antechamber module. The POPC lipid bilayer was described using the Lipid17 force field. The membrane-protein systems were solvated in a rectangular periodic box utilizing the TIP3P water model, maintaining a minimum distance of 15 Å between the solute and the box boundaries along the Z-axis. The systems were neutralized, and the physiological salt concentration was maintained at 0.15 M with NaCl. To remove unfavorable steric clashes, the systems were subjected to multi-step energy minimization, initially applying harmonic restraints on the protein-lipid heavy atoms, followed by unrestrained minimization utilizing steepest descent and conjugate gradient methods. Subsequently, the systems were gradually heated to 300 K under the NVT ensemble, followed by a multi-step equilibration phase under the NPT ensemble to carefully relax the lipid bilayer around the transmembrane domains. The production phase consisted of 200 ns of MD simulation under the NPT ensemble with periodic boundary conditions. Long-range electrostatic interactions were treated with the Particle Mesh Ewald (PME) method, and a cutoff radius of 10 Å was applied for nonbonded interactions, including van der Waals and short-range electrostatics. All bonds involving hydrogen atoms were constrained using the SHAKE algorithm to improve integration efficiency. Temperature was controlled with a Langevin thermostat (ntt = 3) at 300 K with a collision frequency of 2 ps⁻¹. Crucially, to accommodate the physical properties of the lipid bilayer, pressure was controlled using semi-isotropic Berendsen barostat coupling (ntp = 2) at a target pressure of 1 atm and a relaxation time constant of 2 ps. The integration time step for the production runs was set to 2 fs. Energy, trajectory, and restart information were recorded every 1,000 steps. Trajectory and restart files were saved in NetCDF binary format (ioutfm = 1, ntxo = 2) to improve input/output efficiency, and a random seed was set (ig = − 1) to ensure stochasticity across simulations^46^. To explicitly decouple intrinsic transporter dynamics from ligand-induced conformational changes, parallel 200 ns MD simulations were performed for the ligand-free (Apo) states of all selected SLC transporters. The Apo systems were constructed, embedded into the POPC lipid bilayer, and simulated using the exact same protocols, force fields, and environmental parameters as their corresponding holo counterparts.

### Data processing

#### Screening criteria for molecular docking data

The positive control group consisted of compounds experimentally validated to bind to specific SLC family proteins, whereas the negative control group consisted of compounds experimentally validated not to bind to SLC proteins. The binding affinity range of non-binding interactions was determined using the negative controls, while the binding affinity range of binding interactions was established using the positive controls. Based on the mean binding affinity values of the positive and negative control groups, a cutoff was defined to divide all docking results into three categories according to their binding affinity levels. To validate the dynamic stability of the docking predictions while managing computational costs, a stratified random sampling strategy was employed. Rather than arbitrarily selecting top-scoring complexes, the entire dataset of 23,970 pairs was partitioned into three distinct affinity strata (high, medium, and low) using the empirically derived thresholds of the positive and negative controls. By randomly selecting two representative complexes from each predefined stratum using Python’s pseudo-random number generator, we minimized selection bias and ensured that the subsequent MD simulations evaluated the reliability of the docking scoring function across the entire energetic spectrum.

To extract systemic binding patterns from the high-throughput docking dataset, unsupervised hierarchical clustering was performed on the affinity matrix using the seaborn library in Python. The clustering was executed using Ward’s minimum variance method with Euclidean distance metrics applied to both the ligands (rows) and SLC transporters (columns). A diverging color scale was utilized to normalize the visual representation, with the center mathematically anchored to a baseline threshold.

#### Processing of molecular dynamics simulation results

The root mean square deviation (RMSD) refers to the structural deviation of a given conformation at a specific time relative to a reference structure. For the MD simulation results, the RMSD values of the protein throughout the simulation time were calculated, as well as the RMSD values of the small molecules relative to the reference frame within the protein–ligand complexes.

The root mean square fluctuation (RMSF) is an important indicator for analyzing local variations along the protein chain. It describes the structural deviation of a given residue i (or atom) relative to a reference conformation over a period of time, thereby reflecting the degree of atomic flexibility.

The ligand root mean square deviation (RMSD) reflects the binding stability of the ligand within the binding pocket and serves as a complement to the RMSD analysis of flexible binding. Based on the MD simulation results, the RMSD of ligand atoms relative to the binding pocket was calculated over the course of the simulation. The binding pocket was defined as the residues located within 5 Å of the ligand.

The total interaction frequency describes the general spatial contact between the ligand and surrounding protein residues over the simulation period. To evaluate this, the contact frequencies were calculated using the MDAnalysis library by applying a uniform distance cutoff of 4.5 Å to capture all heavy-atom contacts (excluding hydrogen atoms) between the ligand and the target SLC transporter. The interaction frequency for a specific residue was defined as the percentage of frames where at least one heavy atom fell within this threshold. To eliminate transient random collisions, only residues exhibiting an interaction frequency greater than 10.0% were retained for subsequent analysis.

Furthermore, the hydrophobic interaction occupancy provides specific insights into the lipophilic anchoring mechanisms of the investigated UVFs. This occupancy was quantitatively evaluated using the Protein-Ligand Interaction Profiler (ProLIF) package. To optimize computational efficiency, the trajectories were sampled at intervals of 10 frames, and the specific hydrophobic fingerprint was explicitly extracted. Residues were ranked based on the proportion of sampled frames where a valid hydrophobic contact was detected, with a conservative threshold of > 5.0% occupancy applied to identify the core hydrophobic anchors dictating the complex stability.

## Results

### Docking results

**Fig. 1:**
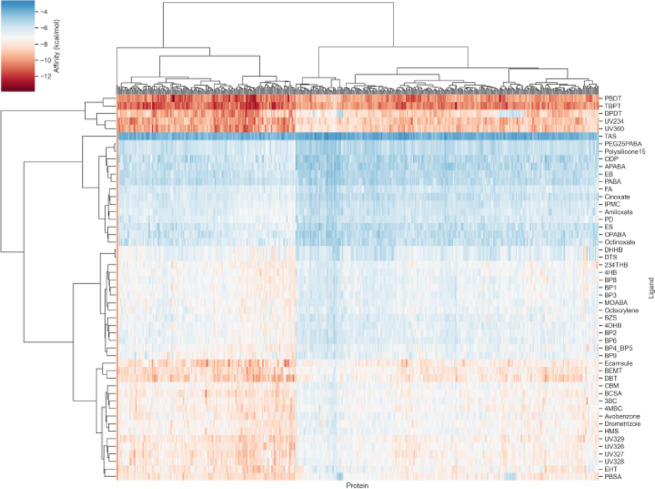
Binding affinities calculated from molecular docking between SLC family proteins and UVF molecules. White tiles indicate the binding affinity of the positive control. The x-axis represents protein file names, and the y-axis represents ligand file names (protein file names and corresponding molecules are listed in Table S1; ligand file names and corresponding molecules are provided in Table S2; detailed data are shown in Table S3). Dendrograms on the top and left axes illustrate the clustering relationships among the SLC transporters and UVF ligands, respectively, revealing distinct target susceptibility patterns and ligand-driven binding strengths.

As shown in Fig. 1, unsupervised hierarchical clustering was applied to the docking affinities of 23,970 UVF-SLC complex pairs to identify systemic binding patterns. The overall binding affinities ranged from − 2.6 to − 13.9 kcal/mol. The clustering dendrograms reveal that binding strength is heavily driven by the intrinsic structural properties of the ligands. A distinct cluster of UVFs, prominently including TBPT, DBT, and LNP (located at the bottom of the y-axis), formed a concentrated red region, exhibiting consistently robust binding affinities across almost the entire SLC proteome. Conversely, a large proportion of smaller or less lipophilic ligands predominantly populated the blue region, indicating generally weaker interactions.

Furthermore, the vertical clustering along the x-axis highlights a pronounced target susceptibility. Specific subgroups of SLC proteins clustered together to form distinct vertical red bands, indicating that these structural families possess binding cavities highly complementary to UVF molecules. Taken together, these results demonstrate that while the lipophilic nature of specific small molecules establishes the baseline binding strength, the distinct architectures of SLC transporter subfamilies concurrently shape the overall affinity landscape.

### Positive and negative controls and selected complexes

**Fig. 2:**
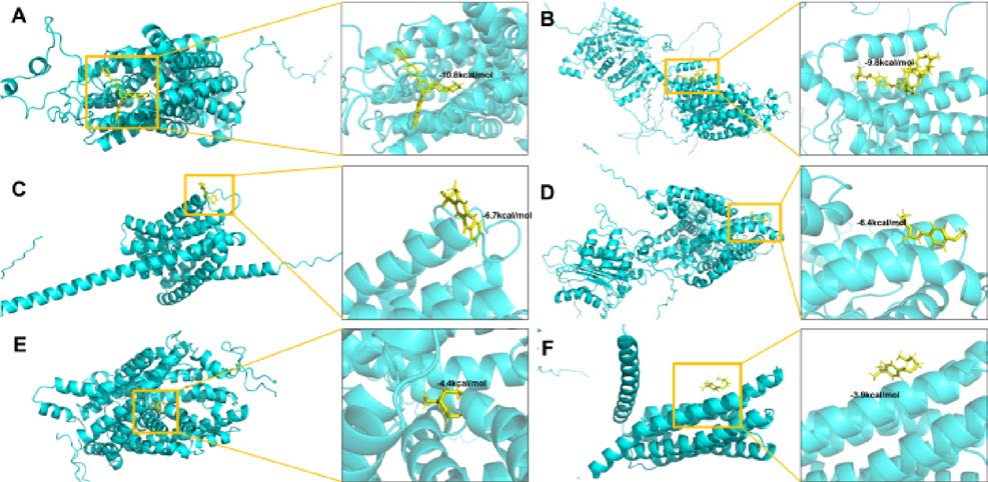
Binding conformations of representative UVF–SLC complexes obtained from molecular docking. (**A**) SLC22A31–TBPT, (**B**) SLC29A4–DBT, (**C**) SLC10A6–BP4_BP5, (**D**) SLC4A9–IPMC, (**E**) SLC6A13–TAS, (**F**) TMEM163–APABA. Proteins are shown in cartoon representation (cyan), and ligands are displayed in stick form (yellow). Enlarged views highlight the binding pockets and local interactions between ligands and surrounding residues. The molecular structures and binding conformations were visualized and rendered using PyMOL v3.1.4.

Negative and positive controls were defined as protein–ligand pairs that had been experimentally validated as non-binding or binding interactions, respectively^47–52^. The mean binding affinity of the positive controls was − 7.17 kcal/mol, whereas that of the negative controls was − 5.3 kcal/mol. Based on these two values, all docking results were divided into three groups, and two complexes were randomly selected from each group, resulting in a total of six representative complexes. The negative controls, positive controls are listed in Table 1 and the selected complexes are summarized in Table 2, which lists the complexes chosen for subsequent MD simulations. The docked conformations of the screened complexes are shown in Fig. 2.

**Table 1:** Positive and negative control protein-ligand complexes for benchmarking docking results.

Classification	Ligand file name	CAS ID	Protein name	Uniprot ID	Affinity (kcal/mol)
Positive control	Methotrexate	59-05-2	SLC19A1	P41440	−7.7
	Uridine	58-96-8	SLC28A1	O00337	−7.0
	Fenoterol	13392-18-2	SLC22A1	O15245	−6.8
Negative control	Glucose	50-99-7	SLC2A13	Q96QE2	−5.7
	vitaminC	50-81-7	SLC2A1	P11166	−5.5
	2-deoxy-D-glucose	154-17-6	SLC5A1	P13866	−4.7

**Table 2:** Complexes selected by random sampling for molecular dynamics simulations.

Classification	Ligand file name	CAS ID	Protein name	Uniprot ID	Affinity (kcal/mol)
High affinity	TBPT	31274-51-8	SLC22A31	A6NKX4	−10.8
	DBT	154702-15-5	SLC29A4	Q7RTT9	−9.8
Medium affinity	BP4_BP5	4065-45-6	SLC10A6	Q3KNW5	−6.7
	IPMC	5466-76-2	SLC4A9	Q96Q91	−6.4
Low affinity	TAS	2174-16-5	SLC6A13	Q9NSD5	−4.4
	APABA	14779-78-3	TMEM163	Q8TC26	−3.9

Since BP5 is the ionic form of BP4, they were grouped together and designated as BP4_BP5.

### Molecular dynamics simulation

#### Protein RMSD analysis

**Fig. 3:**
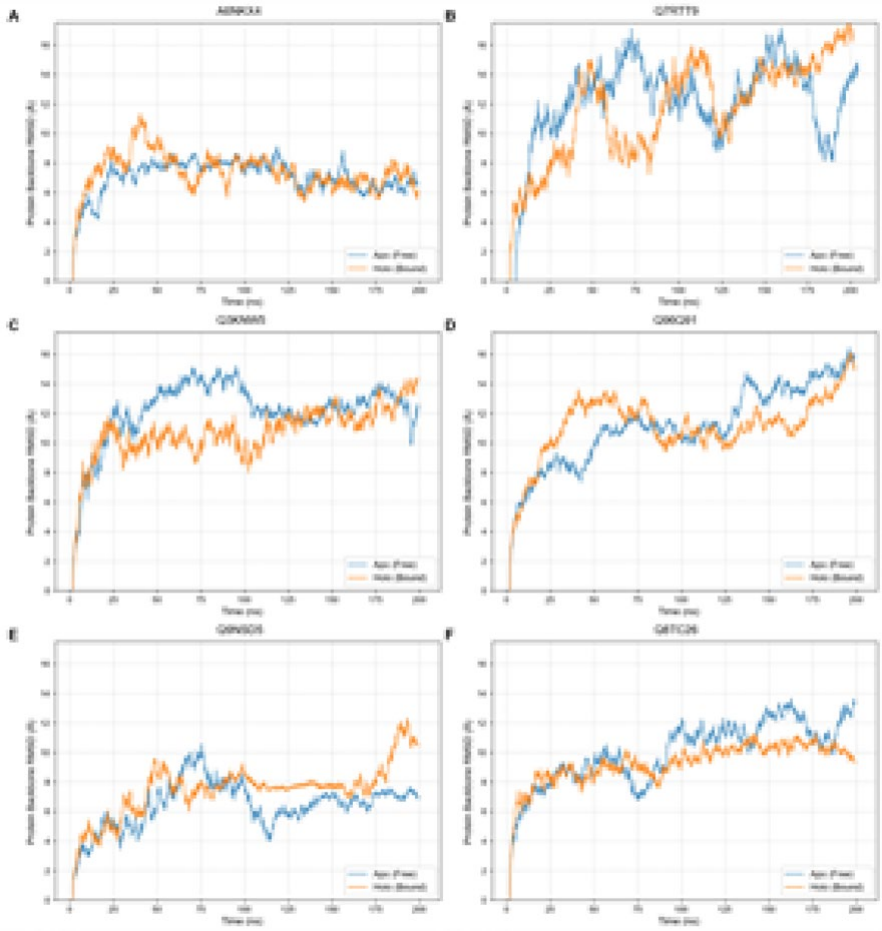
Protein backbone RMSD during the simulations, using the coordinates of frame 0 as the reference. (**A**) RMSD of SLC22A31 with TBPT over 200 ns. (**B**) SLC29A4 with DBT. (**C**) SLC10A6 with BP4_BP5. (**D**) SLC4A9 with IPMC. (**E**) SLC6A13 with TAS. (**F**) TMEM163 with APABA.

To evaluate the macroscopic structural fluctuations of the target proteins during the 200 ns simulations, we monitored the global protein backbone RMSD. In the definitively stable cohort (Fig. 3A and B), the Holo trajectories exhibit noticeable fluctuation dampening relative to their Apo counterparts—most distinctly observed as the suppression of extreme > 15 Å peaks in Fig. 3B. However, their absolute RMSD values remain notably high across the simulation period (ranging from 6 to 14 Å). It is important to note that these high global deviations are primarily intrinsic to the structural relaxation of AlphaFold-predicted models within an explicit lipid bilayer. The initial equilibration naturally involves significant spatial rearrangements of the large, unstructured extra-/intracellular loops and highly flexible terminal domains, rather than a breakdown of the core transmembrane architecture. For the initially ambiguous cohort (Fig. 3C and D), the macroscopic RMSD profiles present contrasting visual trends. Figure 3C displays a visible suppression of the Holo RMSD relative to the Apo state for the majority of the simulation, suggesting a degree of macroscopic stabilization. Conversely, Fig. 3D displays highly intertwined Apo and Holo trajectories throughout the entire 200 ns period, with no significant divergence or continuous dampening observed. Finally, the definitively unstable cohort (Fig. 3E and F) provides robust macroscopic indicators of dynamic instability. Most strikingly, Fig. 3E undergoes a catastrophic trajectory divergence after 150 ns, where the Holo RMSD precipitously surges beyond 12 Å. Similarly, Fig. 3F exhibits chaotic and intertwined profiles with large-scale structural fluctuations, lacking any sustained macroscopic rigidification induced by the ligand.

#### Ligand RMSD Analysis

**Fig. 4:**
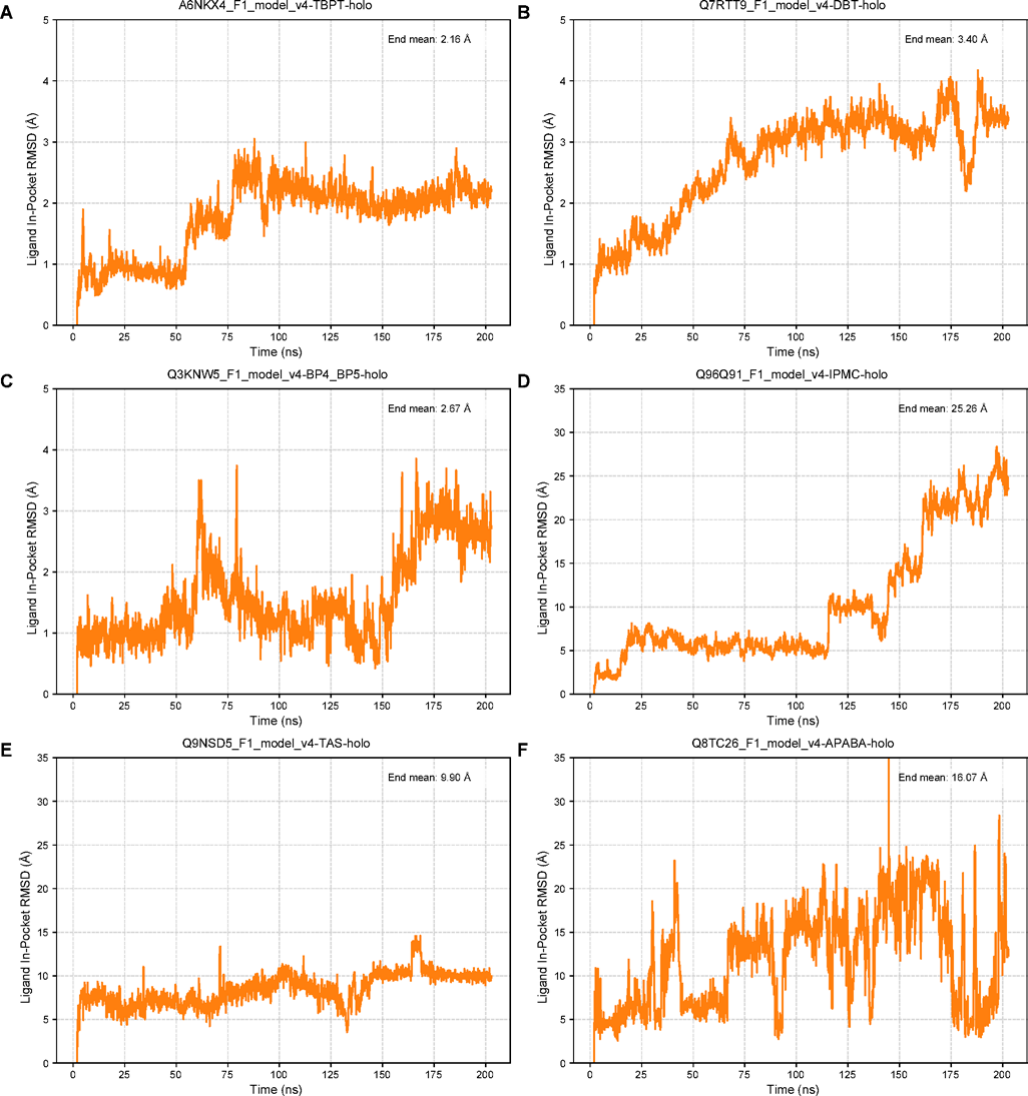
Ligand RMSD during the simulations, calculated relative to the protein binding pocket in frame 0. The binding pocket was defined as protein residues within 5 Å of the ligand. (**A**) Ligand RMSD for SLC22A31 with TBPT over 200 ns. (**B**) SLC29A4 with DBT. (**C**) SLC10A6 with BP4_BP5. (**D**) SLC4A9 with IPMC. (**E**) SLC6A13 with TAS. (**F**) TMEM163 with APABA.

To unambiguously resolve the binding stability and filter out the macroscopic structural noise, we calculated the Ligand In-Pocket RMSD by aligning the trajectories to the initial 5 Å binding pocket backbone (Fig. 4). This highly localized metric effectively isolates the relative translational and rotational motions of the ligand within the active site. The analysis decisively stratifies the complexes into two distinct cohorts.

In the definitively stable binding cohort (Fig. 4A and B), the ligands exhibit remarkable positional retention within the pocket. Despite the global protein flexibility observed earlier, the end-mean In-Pocket RMSD values in Fig. 4A and B are tightly constrained at 2.16 Å and 3.40 Å, respectively. These values fall strictly within the universally accepted threshold for stable binding (< 4.0 Å), proving that these ligands remain firmly anchored to their orthosteric sites throughout the 200 ns simulation. Crucially, the highly localized metric definitively resolves the binding status of the initially ambiguous cohort (Fig. 4C and D). In Fig. 4C, the ligand maintains a highly stable pose with an end-mean RMSD of 2.67 Å, successfully confirming a productive anchoring event. In stark contrast, in Fig. 4D, the ligand undergoes a step-wise escape from the active site, culminating in an extreme end-mean RMSD of 25.26 Å, which effectively invalidates its initial binding pose. Finally, the definitively unstable cohort (Fig. 4E and F) demonstrates catastrophic spatial deviations. Crucially, the violent fluctuations and lack of trajectory convergence observed in these low-affinity panels (E and F) are expected outcomes. They serve as essential internal negative controls, demonstrating that our MD pipeline effectively discriminates and filters out transient surface collisions or false-positive docking predictions. Most instructively, Fig. 4E reveals a delayed unbinding event: the ligand initially persists near the pocket but experiences a definitive dissociation after 150 ns, with its RMSD abruptly jumping to ~ 10 Å, perfectly mirroring the late-stage macroscopic trajectory divergence observed in the global analyses. Similarly, Fig. 4F displays chaotic, high-amplitude fluctuations (end-mean 16.07 Å), indicative of a ligand randomly wandering in the solvent or undergoing non-specific surface collisions.

#### Protein RMSF analysis

**Fig. 5:**
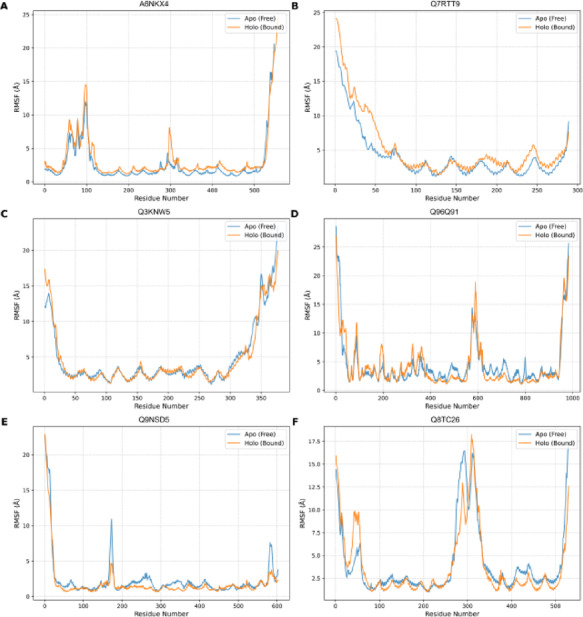
Per-residue RMSF values of proteins during the simulations, referenced to the residue coordinates in frame 0. (**A**) SLC22A31 with TBPT over 200 ns. (**B**) SLC29A4 with DBT over 200 ns. (**C**) SLC10A6 with BP4_BP5 over 200 ns. (**D**) SLC4A9 with IPMC over 200 ns. (**E**) SLC6A13 with TAS over 200 ns. (**F**) TMEM163 with APABA over 200 ns.

To elucidate the residue-level dynamic responses to ligand binding and address the localized origins of the macroscopic backbone deviations, we computed the Root Mean Square Fluctuation (RMSF) for all alpha-carbon atoms (Fig. 5). The RMSF profiles elegantly uncouple localized dynamic adaptations from overall binding stability.

In the definitively stable binding cohort (Fig. 5A and B), the ligands induce target-specific flexibility alterations. Figure 5A demonstrates a generalized elevation in RMSF across multiple loop regions (e.g., residues 50–120 and ~ 300) in the Holo state, reflecting the structural accommodation required to maintain an open pocket conformation. Figure 5B exhibits a pronounced increase in flexibility exclusively at the N-terminal region (residues 1–100) in the Holo state; this localized hyper-flexibility precisely accounts for the anomalously high global backbone RMSD observed previously, confirming that the active site core remains unperturbed. Crucially, the RMSF analysis provides definitive clarification for the initially ambiguous cohort (Fig. 5C and D). Figure 5C displays nearly identical Apo and Holo RMSF profiles with highly restricted fluctuations in the binding core, indicating that the ligand integrates perfectly into the binding pocket without disrupting the protein’s native dynamic network. Conversely, in Fig. 5D, the ligand entirely fails to mitigate the intrinsic structural fluctuations of the protein, leaving the Apo and Holo profiles highly entangled across the entire sequence. For the definitively unstable cohort (Fig. 5E and F), the RMSF data reveal clear signatures of transient or failed interactions. Figure 5E provides a particularly insightful snapshot: despite the ultimate dissociation of the ligand, the Holo state transiently suppresses specific hyper-flexible peaks present in the Apo state (e.g., around residues 170 and 580). Meanwhile, Fig. 5F shows an exacerbation and positional shift of the massive fluctuation in its dominant loop region (residues 280–320), a macroscopic hallmark of non-specific surface clashes.

#### Per-residue interaction profiling analysis

To distinguish productive anchoring from transient surface contact, we assessed the number of “core residues” that maintained a heavy-atom interaction frequency greater than 90% throughout the 200 ns simulations. This metric serves as a direct indicator of the localized binding site’s robustness (Table S4).

A decisive threshold in interaction density was observed between the stable and unstable complexes. In the definitively stable cohort, the ligands are secured by a robust cluster of high-frequency anchors: TBPT-SLC22A31 and DBT-SLC29A4 are stabilized by 8 and 7 core residues (> 90%), respectively. Similarly, the localized stability of BP4_BP5-SLC10A6 is resolved by the presence of 6 core residues, including the absolute 100% occupancy of ILE88 and TRP152. Notably, these high-occupancy residues predominantly localize within the putative central permeation pathways or structurally critical transmembrane helices of the transporters. This high density of persistent contacts suggests a cooperative “energy trap” mechanism that prevents ligand escape but also potentially locks the transporter into an occluded, inactive conformation, thereby competitively impeding the translocation of endogenous substrates.

In sharp contrast, the unstable cohort displays a near-total lack of such high-frequency clusters. IPMC-SLC4A9 possesses zero residues with > 90% frequency, explaining its rapid loss of positional control. While TAS-SLC6A13 and APABA-TMEM163 each retain only 2 isolated high-frequency residues, this sparse anchoring is insufficient to sustain the complex, ultimately leading to the catastrophic dissociations observed in the RMSD trajectories. Within the scope of our screened dataset, these results demonstrate that stable orthosteric binding is a collective, multi-residue event that typically coincides with the formation of a localized interaction network comprising multiple (e.g., 6 or more) high-frequency anchors.

#### Hydrophobic interaction profiling of stable complexes

While the previous analysis identified the overall binding footprint, it is essential to dissect the specific interaction types to elucidate the dominant driving forces governing complex stability. Given the highly lipophilic nature of organic UV filters, hydrophobic networks often provide the primary thermodynamic contribution to their transmembrane binding. Therefore, we quantitatively evaluated the occupancy of hydrophobic contacts over the 200 ns trajectories for the stable complex cohort (Table S5).

In the TBPT–SLC22A31 complex, stable anchoring was heavily dependent on a concentrated hydrophobic core. The aromatic residue PHE367 exhibited a remarkable hydrophobic occupancy of 90.0%, suggesting robust $\:{\uppi\:}-{\uppi\:}$ or hydrophobic stacking with the aromatic rings of TBPT. Additionally, the aliphatic portions of ARG481 (32.0%) and GLN45 (31.5%), along with TRP127 (26.5%), provided supplementary hydrophobic confinement. Together, these residues form a spatially complementary hydrophobic cavity that severely restricts the translational and rotational degrees of freedom of the ligand.

The DBT–SLC29A4 complex further highlighted the necessity of decoupling interaction types. While the total interaction profiling indicated continuous spatial proximity with various residues, the isolated hydrophobic analysis revealed that ILE157 acts as the primary thermodynamic anchor, maintaining a 90.5% hydrophobic occupancy. The aliphatic carbon backbone of ARG280 (77.0%), cooperatively supported by VAL276 (27.0%) and VAL160 (21.5%), tightly locks DBT within the transmembrane core. This persistent hydrophobic network perfectly corroborates the highly restricted In-Pocket RMSD fluctuations observed for this complex. Functionally, as SLC29A4 (PMAT) is heavily involved in the transport of monoamines and organic cations, the permanent occupation of its transmembrane core by DBT suggests a high risk of transport blockade, potentially disrupting local metabolite gradients.

Notably, the BP4_BP5–SLC10A6 complex demonstrated exceptional interaction specificity. TRP152 maintained an absolute 100.0% hydrophobic occupancy throughout the entire simulation, acting as an immovable dynamic barrier. Supported by high-frequency hydrophobic contacts from ILE88 (80.5%) and LEU154 (41.5%), the ligand is effectively captured in a structural “hydrophobic trap”. This specific anchoring mechanism explains how this initially classified medium-affinity system sustains robust structural stability in a dynamic lipid environment. Considering that SLC10A6 functions as a critical transporter for steroidal sulfates^53^, this rigid hydrophobic trapping mechanism provides a compelling atomistic explanation for the endocrine-disrupting potentials widely reported for benzophenone-type UVFs.

Collectively, these findings demonstrate that high-occupancy hydrophobic anchors (> 80%) dictate the persistent conformational states of these UVF-SLC complexes. Beyond merely stabilizing the ligand, these localized hydrophobic networks likely convert transient exposures into prolonged structural occlusions, underscoring the critical role of lipophilicity in mediating the long-lasting binding of these emerging contaminants to human transporters.

## Discussion

This study integrated systematic molecular docking and MD simulations to elucidate the interaction characteristics between representative organic UVFs and SLC transporters, and the dynamic stability of their complexes. The three-dimensional structures of SLC proteins were predicted using AlphaFold, whose applicability to emerging contaminants has been validated in previous studies; for instance, AlphaFold-predicted structures have been employed to perform proteome-wide docking and interaction network analyses of endocrine-disrupting chemicals, demonstrating its reliability in revealing multi-target and multi-pathway mechanisms of pollutant–protein interactions^54^. Building upon this structural platform, we constructed a comprehensive screening database encompassing 51 UVFs and 470 SLC proteins. To the best of our knowledge, this is the first effort to establish a superfamily-wide dataset mapping the transmembrane interaction profiles of UVFs. Compared with previous studies that focused on isolated UVF-SLC interactions^14^, our computational investigation encompasses the entire human SLC superfamily. Given that SLC transporters serve as critical gatekeepers of cellular metabolism and xenobiotic clearance, this broad scope provides essential, systematic support for predicting organ-specific toxicities and metabolic disruptions induced by systemic UVF exposure. Our initial docking revealed binding affinities ranging from approximately − 2.6 to − 13.9 kcal/mol (Fig. 1), establishing a baseline for structural susceptibility. In general, the magnitude of the docking affinity was broadly consistent with dynamic stability under physiological lipid conditions. In certain high-affinity complexes (e.g., DBT–SLC29A4 and TBPT–SLC22A31), ligand RMSD remained within 3 Å throughout the simulations, indicating stable residency within the binding pocket; by contrast, in low-affinity pairings (e.g., APABA–TMEM163 and TAS–SLC6A13), ligand RMSD rose progressively and exceeded 15 Å, suggesting unstable binding or partial/complete dissociation. Overall, these results indicate that while docking binding affinity can provide a highly efficient, coarse proxy for identifying potential targets, MD simulations offer essential complementary evidence for filtering out transient surface contacts and conclusively assessing binding persistence in a dynamic membrane environment.

To enhance the accuracy of molecular docking results, this study employed both negative and positive controls for validation. Based on these controls, the docking results were divided into three categories, which can be preliminarily regarded as strong, moderate, and weak affinities, corresponding respectively to “binding,” “possible binding or non-binding,” and “no binding.” However, because molecular docking inherently relies on static approximations and cannot definitively confirm binding stability under physiological lipid conditions, we systematically advanced representative complexes from each category into rigorous MD simulations for further validation^55^. In this study, molecular dynamics simulations were then performed for each category. The results revealed distinct patterns in the stability of complexes based on their affinity levels. For example, it was shown that complexes with strong affinity consistently formed stable structures (Panels A, B in Figs. 3, 4 and 5), those with weak affinity failed to maintain stable complexes (Panels E, F in Figs. 3, 4 and 5), and those with moderate affinity exhibited mixed behaviors—some stable and some unstable (Panels C, D in Figs. 3, 4 and 5). Crucially, these in silico predictions strongly align with existing macroscopic toxicological data. Among the compounds evaluated, BP4 has well-documented biotoxicological evidence demonstrating acute toxicity in vivo^56^. The fact that BP4 (and its ionic state BP5) emerged in our MD simulations as forming an exceptionally stable, irreversibly anchored complex with SLC10A6 provides compelling, independent validation of our computational framework. These findings indicate that the molecular docking results can preliminarily identify which organic UVFs are capable of binding to SLC proteins, thereby offering a reliable mechanistic basis for their observed systemic toxicities.

Mechanistically, our per-residue interaction profiling (Sect. 3.3.4 and 3.3.5) provides solid atomic-level evidence for this dynamic stability. For stably bound complexes (e.g., DBT–SLC29A4, TBPT–SLC22A31, and BP4/BP5–SLC10A6), persistent ligand residence is dictated by high-occupancy hydrophobic networks rather than random spatial constraints. Specifically, we identified critical hydrophobic anchors—such as PHE367 in SLC22A31, ILE157 in SLC29A4, and TRP152 in SLC10A6—that maintain > 80% interaction occupancy, effectively forming rigid “hydrophobic traps” around the lipophilic UVFs. Such highly specific and durable binding modes likely occlude the natural substrate-binding cavities, severely interfering with normal transmembrane transport by SLCs. This is highly consistent with competitive inhibition mechanisms widely reported for drug–transporter interactions^19,20^. Nevertheless, we emphasize that these proposed inhibitory mechanisms are predictive computational models. Subsequent in vitro transport assays will be required to translate these structural stabilities into confirmed functional disruptions. By contrast, unstable or transient complexes (e.g., TAS–SLC6A13 and APABA–TMEM163) failed to establish such dominant hydrophobic anchors, leading to progressive ligand escape. This suggests that their modes of action might be dominated by fleeting non-specific contacts or indirect modulation of protein conformational dynamics via membrane-phase accumulation^1,14^.

However, several methodological limitations should be noted. First, although employing AlphaFold-predicted structures for docking and simulations is pragmatic when high-resolution structures are unavailable, such models may not fully capture the conformational polymorphism of transporters^31^. Second, it is important to acknowledge that the binding affinities calculated by AutoDock Vina (− 2.6 to − 13.9 kcal/mol) are derived from a semi-empirical scoring function. These scores are approximate and cannot be directly translated into exact experimental binding free energies. In this study, the docking scores were strictly utilized for relative ranking and high-throughput stratification prior to MD simulations, rather than as absolute quantitative indicators of thermodynamic binding strength. The integration of experimentally validated controls helped mitigate this limitation by establishing relative, system-specific empirical thresholds. Moving forward, to overcome the static nature of AlphaFold predictions, employing enhanced sampling techniques (e.g., metadynamics) or utilizing emerging Cryo-EM structures will be crucial for exploring the complete conformational cycle and dynamic state transitions of these transporters. Ultimately, transitioning from in silico predictions to in vitro and in vivo validations—such as surface plasmon resonance (SPR), isothermal titration calorimetry (ITC), or site-directed mutagenesis of the identified high-frequency core residues—will be essential to conclusively validate our proposed atomistic binding mechanisms and lipid-mediated anchoring paradigms.

Overall, this study differentiates docking affinity from dynamic stability, identifies representative complexes with stable binding characteristics (e.g., DBT–SLC29A4 and BP4/BP5–SLC10A6), and shows that the complex stability is primarily governed by local functional group–residue complementarity. By providing standardized computational evidence, our dataset establishes an innovative molecular reference for evaluating UVF–transporter interactions and offers practical guidance for toxicological studies, risk assessment, and regulatory decision-making.

## Conclusion

This study combined molecular docking and MD simulation to comprehensively analyze the interactions between organic UVFs and SLC transporters. The results show that certain organic UVFs (e.g., DBT and BP4/BP5) form stable complexes with specific SLCs (e.g., SLC29A4 and SLC10A6), whereas other compounds (e.g., APABA) exhibit unstable or transient binding. While our computational data reveal distinct interaction and stability patterns, we acknowledge that structural binding does not inherently imply functional transport or direct toxicity. Rather, the present dataset provides a novel molecular benchmark for identifying potential toxicological targets. This standardized in silico resource highlights the necessity for targeted functional assays in the future, ultimately guiding risk assessment and supporting regulatory decision-making regarding organic UVFs exposure.

## Supplementary Information

Below is the link to the electronic supplementary material.

Supplementary Material 1

Supplementary Material 2

## Data Availability

All data supporting the findings of this study are available within the paper and its Supplementary Information.
