# Supplementary material for: Probing the binding stability of organic UV filters to human SLC transporters using AlphaFold and molecular dynamics simulations
Source: Sci Rep. 2026 Jun 5;16:25773. doi: 10.1038/s41598-026-56714-z (PMC13483042; doi:10.1038/s41598-026-56714-z)
Supplement: Supplementary file 1 [file 41598_2026_56714_MOESM1_ESM.pdf]

Probing the Binding Stability of Organic Ultraviolet Filters to Human Solute Carrier Transporters Using AlphaFold and Molecular Dynamics Simulations

Ruikai Xing[1, #], Zhongquan Jiang[1,2, #], Tianyi Wei[1], Jiale Chen[1,3], Lijun Zhang[4], Shuangqing Hu[5], Zhemin Shen[1, *], Tao Yuan[1, *]

[1] Key Laboratory of Environmental Health Impact Assessment of Emerging Contaminants, Ministry of Ecology and Environment, School of Environmental Science and Engineering, Shanghai Jiao Tong University, Shanghai 200240, China

[2] East China Sea Fisheries Research Institute, Chinese Academy of Fishery Sciences, Shanghai, 200090, China

[3] School of Big Data and Artificial Intelligence, Chizhou University, Chizhou 247000, China

[4] Division of Public Health Service and Safety Assessment, Shanghai Municipal Center for Disease Control and Prevention, Shanghai 201107, China

[5] Shanghai Academy of Environmental Sciences, Shanghai 200233, China

[#] These authors contributed equally to this work.

*** Corresponding author:**

Prof. Tao Yuan; Prof. Zhemin Shen

Key Laboratory of Environmental Health Impact Assessment of Emerging Contaminants, Ministry of Ecology and Environment, School of Environmental Science and Engineering, Shanghai Jiao Tong University, Shanghai 200240, China

E-mail: taoyuan@sjtu.edu.cn (T. Yuan); zmshen@sjtu.edu.cn (Z. Shen)

Table S1. Organic UV Filters and Related Information

Family	Compound name	Filename	CAS	Structure	Log Kow
	2,4-Dihydroxybenzophenone	BP1	131-56-6	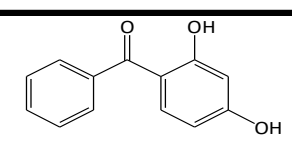	2.19
	2,2',4,4'-Tetrahydroxybenzophenone	BP2	131-55-5	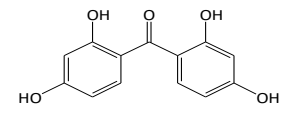	2.78
	2-Hydroxy-4-methoxybenzophenone	BP3	131-57-7	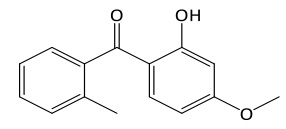	3.79
	2-benzoyl-5-methoxy-1-phenol-4-sulfonic acid	BP4_BP5	4065-45-6	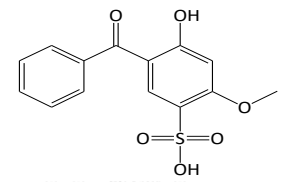	0.37
	2,2'-Dihydroxy-4,4'-dimethoxybenzophenone	BP6	131-54-4	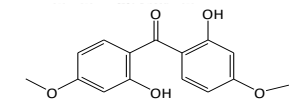	3.9
Benzophenone derivatives	2,2-dihydroxy-4-methoxybenzophenone	BP8	131-53-3	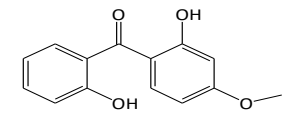	3.82
	2,2'-dihydroxy-4,4'-dimethoxybenzophenone-5,5'-disodium sulfonate disodium salt	BP9	76656-36-5	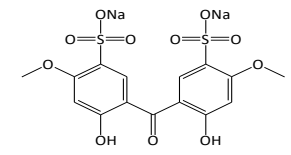	-2.78
	4-Hydroxybenzophenone	4HB	1137-42-4	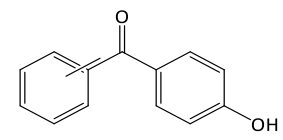	3.07
	4,4'-Dihydroxybenzophenone	4DHB	611-99-4	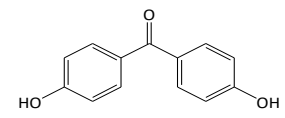	2.19
	Diethylaminohydroxybenzoyl hexyl benzoate	DHHB	302776-68-7	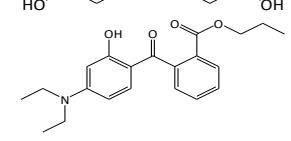	6.54
	2,3,4-Trihydroxybenzophenone	234THB	1143-72-2	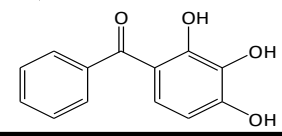	2.91
	2-ethylhexyl 4-(dimethylamino)benzoate	OPABA	21245-02-3	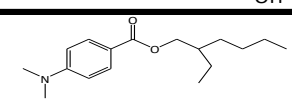	5.77
	Ethyl 4-aminobenzoate	EB	94-09-7	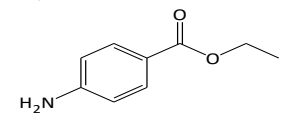	1.86
	Octyl Dimethyl p-aminobenzoate	ODP	58817-05-3	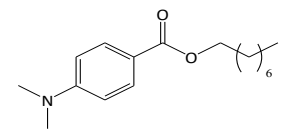	5.84
PABA derivatives	4-Aminobenzoic acid	PABA	150-13-0	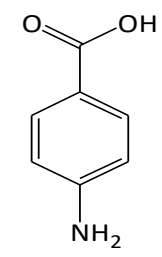	0.83
	PEG-25 PABA	PEG25PABA	116242-27-4	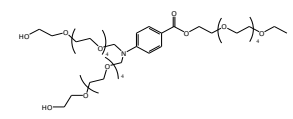	—
	Pentyl 4-(dimethylamino)benzoate	APABA	14779-78-3	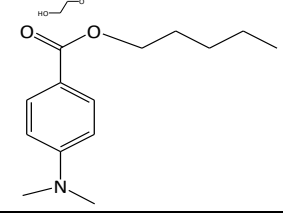	4.37
Anthranilic acid derivatives	menthyl anthranilate	MOABA	134-09-8	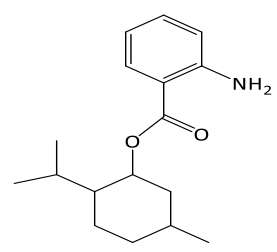	6.28
	3-(4-Methylbenzylidene)-camphor	4MBC	36861-47-9	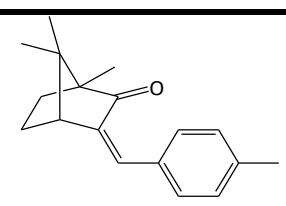	5.92
	(E-E)-3,3'-(1,4-Phenylenedimethylidene)bis(2-oxobornane-10-sulfonic acid)	Ecamsule	92761-26-7	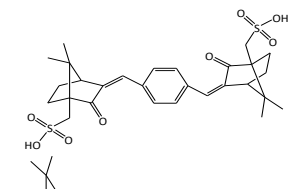	3.83
Camphor derivatives	Camphor Benzalkonium Methosulfate	CBM	52793-97-2	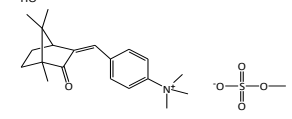	3.11
	4-((4,7,7-Trimethyl-3-oxo-bicyclo(2.2.2)hept-2-ylidene)methyl)benzenes ulfonic acid	BCSA	56039-58-8	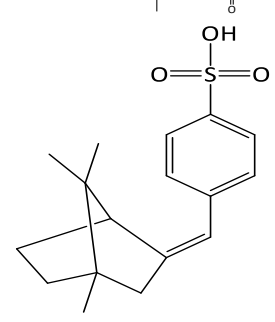	2.22
	3-benzylidene Camphor	3BC	15087-24-8	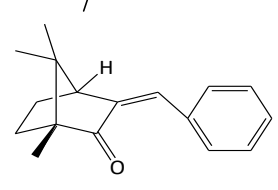	5.37

Benzotriazole derivatives	2-(2H-benzotriazol-2-yl)-4,6-bis(2-phenylpropan-2-yl)phenol	UV234	70321-86-7	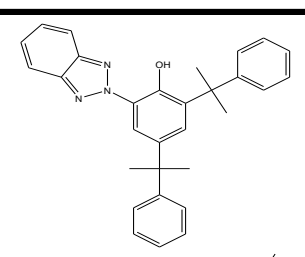	7.67
	2-tert-butyl-6-(5-chloro-2H-benzotriazol-2-yl)-4-methylphenol	UV326	3896-11-5	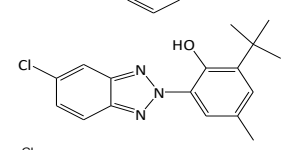	5.55
	2,4-Di-tert-butyl-6-(5-chloro-2H-benzotriazol-2-yl)phenol	UV327	3864-99-1	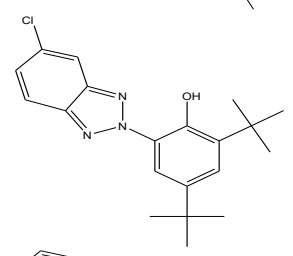	6.91
	2-(2H-benzotriazol-2-yl)-4,6-di-tert-pentylphenol	UV328	25973-55-1	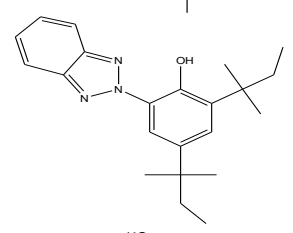	7.25
	2-(2H-benzotriazol-2-yl)-4-(1,1,3,3-tetramethylbutyl)phenol	UV329	3147-75-9	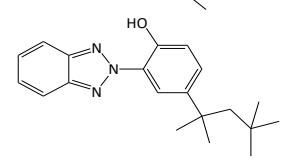	7.29
	Diurethane dimethacrylate	UV360	103597-45-1	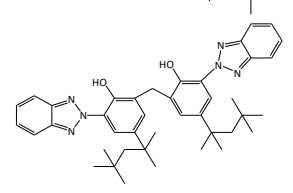	12.46
	2-(2'-Hydroxy-5'-methyl-phenyl)benzotriazole	Drometrizole	2440-22-4	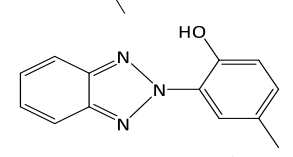	4.31
	DroMetrizde trisiloxane	DTS	155633-54-8	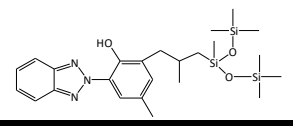	10.82
Salicylic acid derivatives	2-Ethylhexyl salicylate	ES	118-60-5	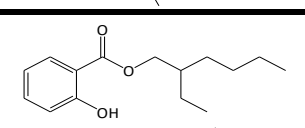	5.97
	Benzoicacid,2-hydroxy-,3,3,5-trimethylcyclohexylester	HMS	118-56-9	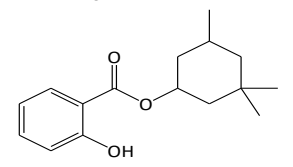	6.16
	2-Hydroxybenzoic acid phenylmethyl ester	BZS	118-58-1	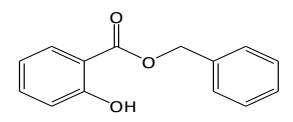	4.31
	Triethanolamine salicylate	TAS	2174-16-5	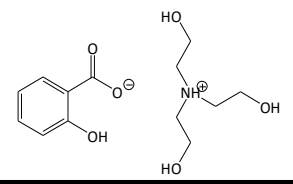	-0.53
Triazine derivatives	Diethylhexyl butamido triazone	DBT	154702-15-5	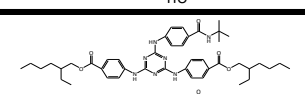	14.03
	Ethylhexyl triazone	EHT	88122-99-0	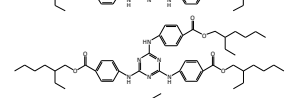	17.05
	Bis-ethylhexyloxyphenol methoxyphenyl triazine	BEMT	187393-00-6	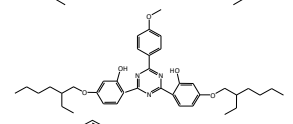	9.29
	3,3'-(1,4-Phenylene)bis(5,6-diphenyl-1,2,4-triazine) （PBDT）	PBDT	55514-22-2	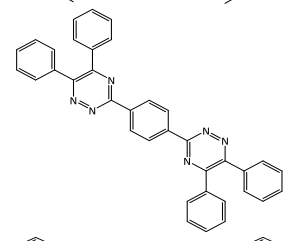	—
	Tris-biphenyl triazine	TBPT	31274-51-8	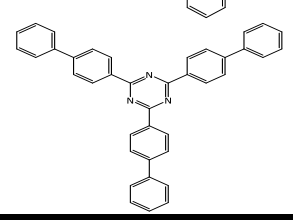	10.38
Cinnamic acid derivatives	2-Ethylhexyl-p-methoxycinnamate	Octinoxate	5466-77-3	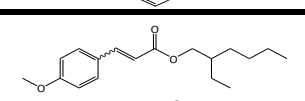	5.8
	Isoamyl 4-methoxycinnamate	Amiloxate	71617-10-2	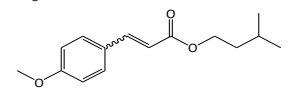	4.33
	Cinoxate	Cinoxate	104-28-9	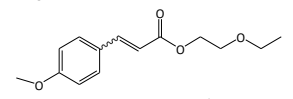	2.65
	isopropyl p-methoxycinnamate	IPMC	5466-76-2	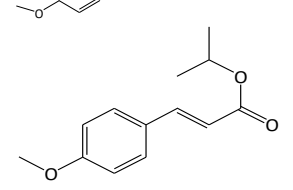	3.35
	4-Hydroxy-3-methoxycinnamic acid	FA	1135-24-6	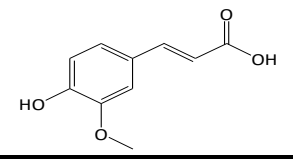	1.51
Dibenzoylmethane derivatives	4-tert-butyl-4'-methoxy-dibenzoylmethane	Avobenzone	70356-09-1	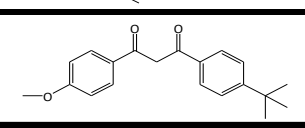	4.51
Benzimidazole derivatives	2-phenyl-1H-benzimidazole-6-sulfonate	PBSA	27503-81-7	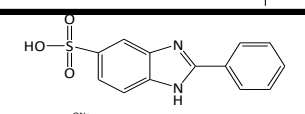	-0.16
	Disodium phenyl dibenzimidazole tetrasulfonate	DPDT	180898-37-7	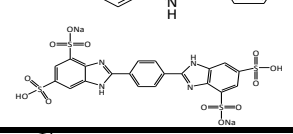	-2
Acrylate derivatives	2-ethylhexyl 2-cyano-3,3-diphenyl-acrylate	Octocrylene	6197-30-4	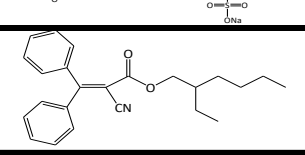	6.88
Benzylidene malonate derivatives	Polysilicone-15	Polysilicone15	207574-74-1	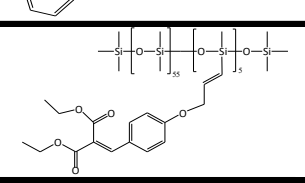	—

Others	1-(3,4-Dimethoxyphenyl)-4,4-dimethyl-1,3-pentanedione	PD	135099-97-7	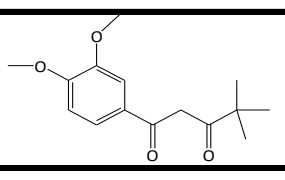	1.61

Table S2. Protein Names and Related Information

UniprotID	Gene	File name	Sequence length	Average pLDDT
Q9HCJ1	ANKH	Q9HCJ1-F1-model_v4	492	84.62 (High)
Q53S99	C2orf83	Q53S99-F1-model_v4	150	54.02 (Low)
Q13286	CLN3	Q13286-F1-model_v4	438	80.83 (High)
Q9NRU3	CNNM1	Q9NRU3-F1-model_v4	951	66.34 (Low)
Q9H8M5	CNNM2	Q9H8M5-F1-model_v4	875	70.24 (High)
Q8NE01	CNNM3	6MN6	707	—
Q6P4Q7	CNNM4	Q6P4Q7-F1-model_v4	775	76.29 (High)
O60931	CTNS	O60931-F1-model_v4	367	88.88 (High)
Q9Y5Y0	FLVCR1	Q9Y5Y0-F1-model_v4	555	78.06 (High)
Q9UPI3	FLVCR2	Q9UPI3-F1-model_v4	526	80.95 (High)
O95202	LETM1	9BA1	739	—
Q2VYF4	LETM2	Q2VYF4-F1-model_v4	491	61.73 (Low)
Q6P1Q0	LETMD1	Q6P1Q0-F1-model_v4	360	80.9 (High)
Q9H0U3	MAGT1	Q9H0U3-F1-model_v4	335	86.45 (High)
Q9H3U5	MFSD1	Q9H3U5-F1-model_v4	465	88.96 (High)
Q14728	MFSD10	Q14728-F1-model_v4	455	91.91 (Very High)
Q6NUT3	MFSD12	Q6NUT3-F1-model_v4	480	84.68 (High)
Q14CX5	MFSD13A	Q14CX5-F1-model_v4	517	81.92 (High)
Q96MC6	MFSD14A	Q96MC6-F1-model_v4	490	78.87 (High)
Q5SR56	MFSD14B	Q5SR56-F1-model_v4	506	77.05 (High)
Q5VZR4	MFSD14C	Q5VZR4-F1-model_v4	150	57 (Low)
Q8NA29	MFSD2A	Q8NA29-F1-model_v4	543	80.48 (High)
A6NFX1	MFSD2B	A6NFX1-F1-model_v4	504	78.67 (High)
Q96ES6	MFSD3	Q96ES6-F1-model_v4	412	90.02 (Very High)
Q8N468	MFSD4A	Q8N468-F1-model_v4	514	76.99 (High)
Q5TF39	MFSD4B	Q5TF39-F1-model_v4	518	78.03 (High)
Q6N075	MFSD5	Q6N075-F1-model_v4	450	88.26 (High)
Q6ZSS7	MFSD6	9MXC	791	—
Q8IWD5	MFSD6L	Q8IWD5-F1-model_v4	586	74.03 (High)
Q8NHS3	MFSD8	Q8NHS3-F1-model_v4	518	83.5 (High)
Q8NBP5	MFSD9	Q8NBP5-F1-model_v4	474	82.37 (High)
Q9Y5U8	MPC1	Q9Y5U8-F1-model_v4	109	91.67 (Very High)
P0DKB6	MPC1L	P0DKB6-F1-model_v4	136	58.2 (Low)
O95563	MPC2	O95563-F1-model_v4	127	89.65 (High)
O75352	MPDU1	O75352-F1-model_v4	247	89.35 (High)
Q9NZJ7	MTCH1	Q9NZJ7-F1-model_v4	389	74.2 (High)
Q9Y6C9	MTCH2	Q9Y6C9-F1-model_v4	303	85.88 (High)
Q7RTP0	NIPA1	Q7RTP0-F1-model_v4	329	84.52 (High)
Q8N8Q9	NIPA2	Q8N8Q9-F1-model_v4	360	83.35 (High)
Q6NVV3	NIPAL1	Q6NVV3-F1-model_v4	410	80.56 (High)
Q6P499	NIPAL3	Q6P499-F1-model_v4	406	75.13 (High)
O15118	NPC1	O15118-F1-model_v4	1278	85.12 (High)
Q9UHC9	NPC1L1	Q9UHC9-F1-model_v4	1359	84.27 (High)
Q04671	OCA2	Q04671-F1-model_v4	838	74.16 (High)
Q9H841	PCON_02445	Q9H841-F1-model_v4	383	78.98 (High)
Q6ZP29	PCON_03420	Q6ZP29-F1-model_v4	291	81.28 (High)
Q9UJS0	PCON_08980	Q9UJS0-F1-model_v4	675	80.56 (High)
Q0D2K0	PCON_10648	Q0D2K0-F1-model_v4	466	72.39 (High)
Q13635	PTCH1	Q13635-F1-model_v4	1447	73.91 (High)
Q9Y6C5	PTCH2	Q9Y6C5-F1-model_v4	1203	78.72 (High)
Q96NR3	PTCHD1	Q96NR3-F1-model_v4	888	85.69 (High)

Q3KNS1	PTCHD3	Q3KNS1-F1-model_v4	767	78.66 (High)
Q6ZW05	PTCHD4	Q6ZW05-F1-model_v4	846	82.64 (High)
Q96AA3	RFT1	Q96AA3-F1-model_v4	541	90.27 (Very High)
Q02094	RHAG	Q02094-F1-model_v4	409	95.09 (Very High)
Q9H310	RHBG	Q9H310-F1-model_v4	441	92.06 (Very High)
P18577	RHCE	P18577-F1-model_v4	417	83.75 (High)
Q9UBD6	RHCG	Q9UBD6-F1-model_v4	479	92.18 (Very High)
Q02161	RHD	Q02161-F1-model_v4	417	85.2 (High)
Q9H9B4	SFXN1	Q9H9B4-F1-model_v4	322	86.01 (High)
Q96NB2	SFXN2	Q96NB2-F1-model_v4	322	85.06 (High)
Q9BWM7	SFXN3	Q9BWM7-F1-model_v4	321	86.64 (High)
Q6P4A7	SFXN4	Q6P4A7-F1-model_v4	337	85.43 (High)
Q8TD22	SFXN5	Q8TD22-F1-model_v4	340	87.77 (High)
Q14973	SLC10A1	Q14973-F1-model_v4	349	83.57 (High)
Q12908	SLC10A2	Q12908-F1-model_v4	348	84.63 (High)
P09131	SLC10A3	P09131-F1-model_v4	477	76.75 (High)
Q96EP9	SLC10A4	Q96EP9-F1-model_v4	437	71.54 (High)
Q5PT55	SLC10A5	Q5PT55-F1-model_v4	438	80.6 (High)
Q3KNW5	SLC10A6	Q3KNW5-F1-model_v4	377	79.66 (High)
Q0GE19	SLC10A7	Q0GE19-F1-model_v4	340	85.65 (High)
P49279	SLC11A1	P49279-F1-model_v4	550	82.69 (High)
P49281	SLC11A2	P49281-F1-model_v4	568	80.85 (High)
Q13621	SLC12A1	Q13621-F1-model_v4	1099	78.21 (High)
P55011	SLC12A2	P55011-F1-model_v4	1212	73.63 (High)
P55017	SLC12A3	P55017-F1-model_v4	1021	81.03 (High)
Q9UP95	SLC12A4	Q9UP95-F1-model_v4	1085	81.63 (High)
Q9H2X9	SLC12A5	Q9H2X9-F1-model_v4	1139	78.63 (High)
Q9UHW9	SLC12A6	Q9UHW9-F1-model_v4	1150	79.84 (High)
Q9Y666	SLC12A7	Q9Y666-F1-model_v4	1083	80.6 (High)
A0AV02	SLC12A8	A0AV02-F1-model_v4	714	69.49 (Low)
Q9BXP2	SLC12A9	Q9BXP2-F1-model_v4	914	81.23 (High)
Q9BZW2	SLC13A1	Q9BZW2-F1-model_v4	595	82.38 (High)
Q13183	SLC13A2	Q13183-F1-model_v4	592	83.29 (High)
Q8WWT9	SLC13A3	Q8WWT9-F1-model_v4	602	81.92 (High)
Q9UKG4	SLC13A4	Q9UKG4-F1-model_v4	626	77.12 (High)
Q86YT5	SLC13A5	Q86YT5-F1-model_v4	568	85.64 (High)
Q13336	SLC14A1	Q13336-F1-model_v4	389	92.9 (Very High)
Q15849	SLC14A2	Q15849-F1-model_v4	920	82.36 (High)
P46059	SLC15A1	P46059-F1-model_v4	708	87.38 (High)
Q16348	SLC15A2	Q16348-F1-model_v4	729	83.51 (High)
Q8IY34	SLC15A3	Q8IY34-F1-model_v4	581	82.23 (High)
Q8N697	SLC15A4	Q8N697-F1-model_v4	577	84.1 (High)
A6NIM6	SLC15A5	A6NIM6-F1-model_v4	579	75.65 (High)
P53985	SLC16A1	P53985-F1-model_v4	500	83.1 (High)
Q8TF71	SLC16A10	Q8TF71-F1-model_v4	515	81.88 (High)
Q8NCK7	SLC16A11	Q8NCK7-F1-model_v4	471	84.31 (High)
Q6ZSM3	SLC16A12	Q6ZSM3-F1-model_v4	516	79.57 (High)
Q7RTY0	SLC16A13	Q7RTY0-F1-model_v4	426	88.08 (High)
Q7RTX9	SLC16A14	Q7RTX9-F1-model_v4	510	80.3 (High)
P36021	SLC16A2	P36021-F1-model_v4	539	80.18 (High)
O15427	SLC16A3	O15427-F1-model_v4	465	83.65 (High)
O15374	SLC16A4	O15374-F1-model_v4	487	79.57 (High)
O15375	SLC16A5	O15375-F1-model_v4	505	80.76 (High)
O15403	SLC16A6	O15403-F1-model_v4	523	76.51 (High)

O60669	SLC16A7	O60669-F1-model_v4	478	84.37 (High)
O95907	SLC16A8	O95907-F1-model_v4	504	77.71 (High)
Q7RTY1	SLC16A9	Q7RTY1-F1-model_v4	509	77.24 (High)
Q14916	SLC17A1	Q14916-F1-model_v4	467	87.34 (High)
O00624	SLC17A2	O00624-F1-model_v4	439	83.92 (High)
O00476	SLC17A3	O00476-F1-model_v4	420	79.05 (High)
Q9Y2C5	SLC17A4	Q9Y2C5-F1-model_v4	497	83.87 (High)
Q9NRA2	SLC17A5	Q9NRA2-F1-model_v4	495	83.78 (High)
Q9P2U8	SLC17A6	Q9P2U8-F1-model_v4	582	77.88 (High)
Q9P2U7	SLC17A7	Q9P2U7-F1-model_v4	560	80.1 (High)
Q8NDX2	SLC17A8	Q8NDX2-F1-model_v4	589	78.1 (High)
Q9BYT1	SLC17A9	Q9BYT1-F1-model_v4	436	88.18 (High)
P54219	SLC18A1	P54219-F1-model_v4	525	76.88 (High)
Q05940	SLC18A2	Q05940-F1-model_v4	514	78.59 (High)
Q16572	SLC18A3	Q16572-F1-model_v4	532	76.58 (High)
Q6NT16	SLC18B1	Q6NT16-F1-model_v4	456	84.51 (High)
P41440	SLC19A1	P41440-F1-model_v4	591	72.48 (High)
O60779	SLC19A2	O60779-F1-model_v4	497	81.11 (High)
Q9BZV2	SLC19A3	Q9BZV2-F1-model_v4	496	81.79 (High)
P43005	SLC1A1	P43005-F1-model_v4	524	80.4 (High)
P43004	SLC1A2	P43004-F1-model_v4	574	77.84 (High)
P43003	SLC1A3	P43003-F1-model_v4	542	81.96 (High)
P43007	SLC1A4	P43007-F1-model_v4	532	80.63 (High)
Q15758	SLC1A5	Q15758-F1-model_v4	541	79.02 (High)
P48664	SLC1A6	P48664-F1-model_v4	564	80.15 (High)
O00341	SLC1A7	O00341-F1-model_v4	560	77.82 (High)
Q8WUM9	SLC20A1	Q8WUM9-F1-model_v4	679	70.23 (High)
Q08357	SLC20A2	Q08357-F1-model_v4	652	72.92 (High)
O15245	SLC22A1	O15245-F1-model_v4	554	84.96 (High)
Q63ZE4	SLC22A10	Q63ZE4-F1-model_v4	541	87.13 (High)
Q9NSA0	SLC22A11	Q9NSA0-F1-model_v4	550	87.59 (High)
Q96S37	SLC22A12	Q96S37-F1-model_v4	553	86.18 (High)
Q9Y226	SLC22A13	Q9Y226-F1-model_v4	551	84.75 (High)
Q9Y267	SLC22A14	Q9Y267-F1-model_v4	594	81.12 (High)
Q8IZD6	SLC22A15	Q8IZD6-F1-model_v4	547	82.77 (High)
Q86VW1	SLC22A16	Q86VW1-F1-model_v4	577	84.88 (High)
Q8WUG5	SLC22A17	Q8WUG5-F1-model_v4	538	72.99 (High)
Q96BI1	SLC22A18	Q96BI1-F1-model_v4	424	86.52 (High)
O15244	SLC22A2	O15244-F1-model_v4	555	83.7 (High)
A6NK97	SLC22A20P	A6NK97-F1-model_v4	555	86.38 (High)
A1A5C7	SLC22A23	A1A5C7-F1-model_v4	686	72.11 (High)
Q8N4F4	SLC22A24	Q8N4F4-F1-model_v4	552	87.64 (High)
Q6T423	SLC22A25	Q6T423-F1-model_v4	547	86.91 (High)
O75751	SLC22A3	O75751-F1-model_v4	556	83.66 (High)
A6NKX4	SLC22A31	A6NKX4-F1-model_v4	556	78.09 (High)
Q9H015	SLC22A4	Q9H015-F1-model_v4	551	85.65 (High)
O76082	SLC22A5	O76082-F1-model_v4	557	86.69 (High)
Q4U2R8	SLC22A6	Q4U2R8-F1-model_v4	563	82.49 (High)
Q9Y694	SLC22A7	Q9Y694-F1-model_v4	548	84.92 (High)
Q8TCC7	SLC22A8	Q8TCC7-F1-model_v4	542	85.56 (High)
Q8IVM8	SLC22A9	Q8IVM8-F1-model_v4	553	85.58 (High)
Q9UHI7	SLC23A1	Q9UHI7-F1-model_v4	598	82.01 (High)
Q9UGH3	SLC23A2	Q9UGH3-F1-model_v4	650	77.3 (High)
Q6PIS1	SLC23A3	Q6PIS1-F1-model_v4	610	76.2 (High)

O60721	SLC24A1	O60721-F1-model_v4	1099	54.84 (Low)
Q9UI40	SLC24A2	Q9UI40-F1-model_v4	661	66.82 (Low)
Q9HC58	SLC24A3	Q9HC58-F1-model_v4	644	70.33 (High)
Q8NFF2	SLC24A4	Q8NFF2-F1-model_v4	622	70.53 (High)
Q71RS6	SLC24A5	Q71RS6-F1-model_v4	500	76 (High)
P53007	SLC25A1	P53007-F1-model_v4	311	74.73 (High)
Q9UBX3	SLC25A10	Q9UBX3-F1-model_v4	287	72.4 (High)
Q02978	SLC25A11	Q02978-F1-model_v4	314	85.89 (High)
O75746	SLC25A12	O75746-F1-model_v4	678	81.76 (High)
O95258	SLC25A14	O95258-F1-model_v4	325	77.4 (High)
Q9Y619	SLC25A15	Q9Y619-F1-model_v4	301	86.17 (High)
P16260	SLC25A16	P16260-F1-model_v4	332	83.48 (High)
O43808	SLC25A17	O43808-F1-model_v4	307	85.96 (High)
Q9H1K4	SLC25A18	Q9H1K4-F1-model_v4	315	74.04 (High)
Q9HC21	SLC25A19	Q9HC21-F1-model_v4	320	84.66 (High)
Q9BXI2	SLC25A2	Q9BXI2-F1-model_v4	301	88.44 (High)
O43772	SLC25A20	O43772-F1-model_v4	301	88.06 (High)
Q9BQT8	SLC25A21	Q9BQT8-F1-model_v4	299	89.1 (High)
Q9H936	SLC25A22	Q9H936-F1-model_v4	323	76.52 (High)
Q9BV35	SLC25A23	Q9BV35-F1-model_v4	468	79.12 (High)
Q6NUK1	SLC25A24	Q6NUK1-F1-model_v4	477	82.31 (High)
Q6KCM7	SLC25A25	Q6KCM7-F1-model_v4	469	73.43 (High)
Q70HW3	SLC25A26	Q70HW3-F1-model_v4	274	86.41 (High)
O95847	SLC25A27	O95847-F1-model_v4	323	81.92 (High)
Q96A46	SLC25A28	Q96A46-F1-model_v4	364	72.68 (High)
Q8N8R3	SLC25A29	Q8N8R3-F1-model_v4	303	79.94 (High)
Q00325	SLC25A3	Q00325-F1-model_v4	362	81.28 (High)
Q5SVS4	SLC25A30	Q5SVS4-F1-model_v4	291	81.45 (High)
Q9H0C2	SLC25A31	Q9H0C2-F1-model_v4	315	90.08 (Very High)
Q9H2D1	SLC25A32	Q9H2D1-F1-model_v4	315	85.52 (High)
Q9BSK2	SLC25A33	Q9BSK2-F1-model_v4	321	84.25 (High)
Q6PIV7	SLC25A34	Q6PIV7-F1-model_v4	304	84.07 (High)
Q3KQZ1	SLC25A35	Q3KQZ1-F1-model_v4	300	89.25 (High)
Q96CQ1	SLC25A36	Q96CQ1-F1-model_v4	311	86.85 (High)
Q9NYZ2	SLC25A37	Q9NYZ2-F1-model_v4	338	76.16 (High)
Q96DW6	SLC25A38	Q96DW6-F1-model_v4	304	83.47 (High)
Q9BZJ4	SLC25A39	Q9BZJ4-F1-model_v4	359	72.84 (High)
P12235	SLC25A4	P12235-F1-model_v4	298	92.31 (Very High)
Q8TBP6	SLC25A40	Q8TBP6-F1-model_v4	338	77.35 (High)
Q8N5S1	SLC25A41	Q8N5S1-F1-model_v4	370	71.97 (High)
Q86VD7	SLC25A42	Q86VD7-F1-model_v4	318	90.53 (Very High)
Q8WUT9	SLC25A43	Q8WUT9-F1-model_v4	341	88.07 (High)
Q96H78	SLC25A44	Q96H78-F1-model_v4	314	81.41 (High)
Q8N413	SLC25A45	Q8N413-F1-model_v4	288	79.04 (High)
Q96AG3	SLC25A46	Q96AG3-F1-model_v4	418	73.91 (High)
Q6Q0C1	SLC25A47	Q6Q0C1-F1-model_v4	308	83.23 (High)
Q6ZT89	SLC25A48	Q6ZT89-F1-model_v4	311	74.9 (High)
P05141	SLC25A5	P05141-F1-model_v4	298	91.38 (Very High)
Q9H1U9	SLC25A51	Q9H1U9-F1-model_v4	297	82.55 (High)
Q3SY17	SLC25A52	Q3SY17-F1-model_v4	297	81.55 (High)
Q5H9E4	SLC25A53	Q5H9E4-F1-model_v4	307	85.85 (High)
P12236	SLC25A6	P12236-F1-model_v4	298	91.74 (Very High)
Q9H2B4	SLC26A1	Q9H2B4-F1-model_v4	701	82.51 (High)
Q8NG04	SLC26A10	Q8NG04-F1-model_v4	563	76.93 (High)

Q86WA9	SLC26A11	Q86WA9-F1-model_v4	606	87.12 (High)
P50443	SLC26A2	P50443-F1-model_v4	739	81 (High)
P40879	SLC26A3	P40879-F1-model_v4	764	84.83 (High)
O43511	SLC26A4	O43511-F1-model_v4	780	81.49 (High)
P58743	SLC26A5	P58743-F1-model_v4	744	83.51 (High)
Q9BXS9	SLC26A6	Q9BXS9-F1-model_v4	759	81.84 (High)
Q8TE54	SLC26A7	Q8TE54-F1-model_v4	656	80.31 (High)
Q96RN1	SLC26A8	Q96RN1-F1-model_v4	970	69.05 (Low)
Q7LBE3	SLC26A9	Q7LBE3-F1-model_v4	791	79.33 (High)
Q6PCB7	SLC27A1	Q6PCB7-F1-model_v4	646	90.8 (Very High)
O14975	SLC27A2	O14975-F1-model_v4	620	92.54 (Very High)
Q5K4L6	SLC27A3	Q5K4L6-F1-model_v4	683	85.78 (High)
Q6P1M0	SLC27A4	Q6P1M0-F1-model_v4	643	90.63 (Very High)
Q9Y2P5	SLC27A5	Q9Y2P5-F1-model_v4	690	86.73 (High)
Q9Y2P4	SLC27A6	Q9Y2P4-F1-model_v4	619	90.79 (Very High)
O00337	SLC28A1	O00337-F1-model_v4	649	82.19 (High)
O43868	SLC28A2	O43868-F1-model_v4	658	81.89 (High)
Q9HAS3	SLC28A3	Q9HAS3-F1-model_v4	691	80.33 (High)
Q99808	SLC29A1	Q99808-F1-model_v4	456	87.41 (High)
Q14542	SLC29A2	Q14542-F1-model_v4	456	84.09 (High)
Q9BZD2	SLC29A3	Q9BZD2-F1-model_v4	475	81.86 (High)
Q7RTT9	SLC29A4	Q7RTT9-F1-model_v4	530	74.84 (High)
P11166	SLC2A1	P11166-F1-model_v4	492	89.58 (High)
O95528	SLC2A10	O95528-F1-model_v4	541	74.86 (High)
Q9BYW1	SLC2A11	Q9BYW1-F1-model_v4	496	89.01 (High)
Q8TD20	SLC2A12	Q8TD20-F1-model_v4	617	74.9 (High)
Q96QE2	SLC2A13	Q96QE2-F1-model_v4	648	80.04 (High)
Q8TDB8	SLC2A14	Q8TDB8-F1-model_v4	520	87.17 (High)
P11168	SLC2A2	P11168-F1-model_v4	524	86.69 (High)
P11169	SLC2A3	P11169-F1-model_v4	496	90.44 (Very High)
P14672	SLC2A4	P14672-F1-model_v4	509	88.58 (High)
P22732	SLC2A5	P22732-F1-model_v4	501	90.48 (Very High)
Q9UGQ3	SLC2A6	Q9UGQ3-F1-model_v4	507	81.56 (High)
Q6PXP3	SLC2A7	Q6PXP3-F1-model_v4	512	87.65 (High)
Q9NY64	SLC2A8	Q9NY64-F1-model_v4	477	84.71 (High)
Q9NRM0	SLC2A9	Q9NRM0-F1-model_v4	540	82.55 (High)
Q9Y6M5	SLC30A1	8XM6	507	—
Q6XR72	SLC30A10	Q6XR72-F1-model_v4	485	67.74 (Low)
Q9BRI3	SLC30A2	Q9BRI3-F1-model_v4	323	76.69 (High)
Q99726	SLC30A3	Q99726-F1-model_v4	388	76.24 (High)
O14863	SLC30A4	O14863-F1-model_v4	429	72.7 (High)
Q8TAD4	SLC30A5	Q8TAD4-F1-model_v4	765	77.55 (High)
Q6NXT4	SLC30A6	Q6NXT4-F1-model_v4	461	71.55 (High)
Q8NEW0	SLC30A7	Q8NEW0-F1-model_v4	376	75.98 (High)
Q8IWU4	SLC30A8	Q8IWU4-F1-model_v4	369	81.55 (High)
Q6PML9	SLC30A9	Q6PML9-F1-model_v4	568	74.98 (High)
O15431	SLC31A1	O15431-F1-model_v4	190	71.4 (High)
O15432	SLC31A2	O15432-F1-model_v4	143	72.41 (High)
Q9H598	SLC32A1	Q9H598-F1-model_v4	525	77.63 (High)
O00400	SLC33A1	O00400-F1-model_v4	549	82.12 (High)
Q06495	SLC34A1	Q06495-F1-model_v4	639	72.3 (High)
O95436	SLC34A2	O95436-F1-model_v4	690	72.64 (High)
Q8N130	SLC34A3	Q8N130-F1-model_v4	599	75.51 (High)
P78382	SLC35A1	P78382-F1-model_v4	337	87.9 (High)

P78381	SLC35A2	P78381-F1-model_v4	396	79.25 (High)
Q9Y2D2	SLC35A3	Q9Y2D2-F1-model_v4	325	87.98 (High)
L0R6Q1	SLC35A4	L0R6Q1-F1-model_v4	103	63.18 (Low)
Q96G79	SLC35A4	Q96G79-F1-model_v4	324	85.24 (High)
Q9BS91	SLC35A5	Q9BS91-F1-model_v4	424	76.66 (High)
P78383	SLC35B1	P78383-F1-model_v4	322	86.04 (High)
Q8TB61	SLC35B2	Q8TB61-F1-model_v4	432	80.18 (High)
Q9H1N7	SLC35B3	Q9H1N7-F1-model_v4	401	77.58 (High)
Q969S0	SLC35B4	Q969S0-F1-model_v4	331	86.5 (High)
Q96A29	SLC35C1	Q96A29-F1-model_v4	364	81.79 (High)
Q9NQQ7	SLC35C2	Q9NQQ7-F1-model_v4	365	83.16 (High)
Q9NTN3	SLC35D1	Q9NTN3-F1-model_v4	355	79.63 (High)
Q76EJ3	SLC35D2	Q76EJ3-F1-model_v4	337	85.37 (High)
Q5M8T2	SLC35D3	Q5M8T2-F1-model_v4	416	76.25 (High)
Q96K37	SLC35E1	Q96K37-F1-model_v4	410	74.19 (High)
P0CK97	SLC35E2A	P0CK97-F1-model_v4	266	52.88 (Low)
P0CK96	SLC35E2B	P0CK96-F1-model_v4	405	74.44 (High)
Q7Z769	SLC35E3	Q7Z769-F1-model_v4	313	90.16 (Very High)
Q6ICL7	SLC35E4	Q6ICL7-F1-model_v4	350	81.16 (High)
Q5T1Q4	SLC35F1	Q5T1Q4-F1-model_v4	408	78.08 (High)
Q8IXU6	SLC35F2	Q8IXU6-F1-model_v4	374	82.17 (High)
Q8IY50	SLC35F3	Q8IY50-F1-model_v4	421	74.99 (High)
A4IF30	SLC35F4	A4IF30-F1-model_v4	521	66.84 (Low)
Q8WV83	SLC35F5	Q8WV83-F1-model_v4	523	73.22 (High)
Q8N357	SLC35F6	Q8N357-F1-model_v4	371	84.37 (High)
Q2M3R5	SLC35G1	Q2M3R5-F1-model_v4	365	80.05 (High)
Q8TBE7	SLC35G2	Q8TBE7-F1-model_v4	412	77.36 (High)
Q8N808	SLC35G3	Q8N808-F1-model_v4	338	85.73 (High)
P0C7Q5	SLC35G4	P0C7Q5-F1-model_v4	338	84.44 (High)
Q96KT7	SLC35G5	Q96KT7-F1-model_v4	338	84.27 (High)
P0C7Q6	SLC35G6	P0C7Q6-F1-model_v4	338	83.11 (High)
Q7Z2H8	SLC36A1	Q7Z2H8-F1-model_v4	476	84.75 (High)
Q495M3	SLC36A2	Q495M3-F1-model_v4	483	84.05 (High)
Q495N2	SLC36A3	Q495N2-F1-model_v4	470	84.7 (High)
Q6YBV0	SLC36A4	Q6YBV0-F1-model_v4	504	81.65 (High)
P57057	SLC37A1	P57057-F1-model_v4	533	79.8 (High)
Q8TED4	SLC37A2	Q8TED4-F1-model_v4	501	84.32 (High)
Q8NCC5	SLC37A3	Q8NCC5-F1-model_v4	494	84.48 (High)
O43826	SLC37A4	O43826-F1-model_v4	429	82.76 (High)
Q9H2H9	SLC38A1	Q9H2H9-F1-model_v4	487	79.93 (High)
Q9HBR0	SLC38A10	Q9HBR0-F1-model_v4	1119	54.33 (Low)
Q08AI6	SLC38A11	Q08AI6-F1-model_v4	406	88.72 (High)
Q96QD8	SLC38A2	Q96QD8-F1-model_v4	506	79.18 (High)
Q99624	SLC38A3	Q99624-F1-model_v4	504	78.56 (High)
Q969I6	SLC38A4	Q969I6-F1-model_v4	547	75.12 (High)
Q8WUX1	SLC38A5	Q8WUX1-F1-model_v4	472	79.96 (High)
Q8IZM9	SLC38A6	Q8IZM9-F1-model_v4	456	84.49 (High)
Q9NVC3	SLC38A7	Q9NVC3-F1-model_v4	462	83.81 (High)
A6NNN8	SLC38A8	A6NNN8-F1-model_v4	435	84.07 (High)
Q8NBW4	SLC38A9	Q8NBW4-F1-model_v4	561	77.09 (High)
Q9NY26	SLC39A1	Q9NY26-F1-model_v4	324	80.38 (High)
Q9ULF5	SLC39A10	Q9ULF5-F1-model_v4	831	59.82 (Low)
Q8N1S5	SLC39A11	Q8N1S5-F1-model_v4	342	76.19 (High)
Q504Y0	SLC39A12	Q504Y0-F1-model_v4	691	69.55 (Low)

Q96H72	SLC39A13	Q96H72-F1-model_v4	371	75.31 (High)
Q15043	SLC39A14	Q15043-F1-model_v4	492	72.77 (High)
Q9NP94	SLC39A2	Q9NP94-F1-model_v4	309	82.46 (High)
Q9BRY0	SLC39A3	Q9BRY0-F1-model_v4	314	81.31 (High)
Q6P5W5	SLC39A4	Q6P5W5-F1-model_v4	647	74.73 (High)
Q6ZMH5	SLC39A5	7YF2	540	—
Q13433	SLC39A6	Q13433-F1-model_v4	755	61.35 (Low)
Q92504	SLC39A7	8GZE	469	—
Q9C0K1	SLC39A8	Q9C0K1-F1-model_v4	460	73.44 (High)
Q9NUM3	SLC39A9	Q9NUM3-F1-model_v4	307	79.8 (High)
Q07837	SLC3A1	Q07837-F1-model_v4	685	87.32 (High)
P08195	SLC3A2	P08195-F1-model_v4	630	78.85 (High)
Q9NP59	SLC40A1	Q9NP59-F1-model_v4	571	80.52 (High)
Q8IVJ1	SLC41A1	Q8IVJ1-F1-model_v4	513	77.82 (High)
Q96JW4	SLC41A2	Q96JW4-F1-model_v4	573	73.29 (High)
Q96GZ6	SLC41A3	Q96GZ6-F1-model_v4	507	79.55 (High)
O75387	SLC43A1	O75387-F1-model_v4	559	79.38 (High)
Q8N370	SLC43A2	Q8N370-F1-model_v4	569	78.42 (High)
Q8NBI5	SLC43A3	Q8NBI5-F1-model_v4	491	81.96 (High)
Q8WWI5	SLC44A1	Q8WWI5-F1-model_v4	657	83.05 (High)
Q8IWA5	SLC44A2	Q8IWA5-F1-model_v4	706	84.47 (High)
Q8N4M1	SLC44A3	Q8N4M1-F1-model_v4	653	80.29 (High)
Q53GD3	SLC44A4	Q53GD3-F1-model_v4	710	83.45 (High)
Q8NCS7	SLC44A5	Q8NCS7-F1-model_v4	719	82.47 (High)
Q9Y2W3	SLC45A1	Q9Y2W3-F1-model_v4	782	64.98 (Low)
Q9UMX9	SLC45A2	Q9UMX9-F1-model_v4	530	77.66 (High)
Q96JT2	SLC45A3	Q96JT2-F1-model_v4	553	76.83 (High)
Q5BKX6	SLC45A4	Q5BKX6-F1-model_v4	768	65.55 (Low)
Q96NT5	SLC46A1	Q96NT5-F1-model_v4	459	83.62 (High)
Q9BY10	SLC46A2	Q9BY10-F1-model_v4	475	80.88 (High)
Q7Z3Q1	SLC46A3	Q7Z3Q1-F1-model_v4	461	86.83 (High)
Q96FL8	SLC47A1	Q96FL8-F1-model_v4	570	86.19 (High)
Q86VL8	SLC47A2	Q86VL8-F1-model_v4	602	81.97 (High)
Q6P1K1	SLC48A1	Q6P1K1-F1-model_v4	146	87.63 (High)
Q6UXD7	SLC49A3	Q6UXD7-F1-model_v4	560	74.36 (High)
Q96SL1	SLC49A4	Q96SL1-F1-model_v4	478	83.87 (High)
P02730	SLC4A1	P02730-F1-model_v4	911	81.42 (High)
Q6U841	SLC4A10	Q6U841-F1-model_v4	1118	71.32 (High)
Q8NBS3	SLC4A11	Q8NBS3-F1-model_v4	891	72.74 (High)
P04920	SLC4A2	8GV8	1241	—
P48751	SLC4A3	8Y85	1232	—
Q9Y6R1	SLC4A4	Q9Y6R1-F1-model_v4	1079	70.46 (High)
Q9BY07	SLC4A5	Q9BY07-F1-model_v4	1137	68.4 (Low)
Q9Y6M7	SLC4A7	Q9Y6M7-F1-model_v4	1214	67.05 (Low)
Q2Y0W8	SLC4A8	Q2Y0W8-F1-model_v4	1093	72.62 (High)
Q96Q91	SLC4A9	Q96Q91-F1-model_v4	983	72.38 (High)
Q9BRV3	SLC50A1	Q9BRV3-F1-model_v4	221	89.82 (High)
Q86UW1	SLC51A	Q86UW1-F1-model_v4	340	82.86 (High)
Q9NWF4	SLC52A1	Q9NWF4-F1-model_v4	448	83.81 (High)
Q9HAB3	SLC52A2	Q9HAB3-F1-model_v4	445	83 (High)
Q9NQ40	SLC52A3	Q9NQ40-F1-model_v4	469	80.15 (High)
P13866	SLC5A1	P13866-F1-model_v4	664	83.99 (High)
A0PJK1	SLC5A10	A0PJK1-F1-model_v4	596	87.85 (High)
Q8WWX8	SLC5A11	Q8WWX8-F1-model_v4	675	81.9 (High)

Q1EHB4	SLC5A12	Q1EHB4-F1-model_v4	618	84.81 (High)
P31639	SLC5A2	P31639-F1-model_v4	672	83.49 (High)
P53794	SLC5A3	P53794-F1-model_v4	718	79.14 (High)
Q9NY91	SLC5A4	Q9NY91-F1-model_v4	659	84.82 (High)
Q92911	SLC5A5	Q92911-F1-model_v4	643	81.32 (High)
Q9Y289	SLC5A6	Q9Y289-F1-model_v4	635	79.74 (High)
Q9GZV3	SLC5A7	Q9GZV3-F1-model_v4	580	83.5 (High)
Q8N695	SLC5A8	Q8N695-F1-model_v4	610	85.33 (High)
Q2M3M2	SLC5A9	Q2M3M2-F1-model_v4	681	82.51 (High)
A1A4F0	SLC66A1L	A1A4F0-F1-model_v4	135	66.24 (Low)
Q8N2U9	SLC66A2	Q8N2U9-F1-model_v4	271	85.56 (High)
Q8N755	SLC66A3	Q8N755-F1-model_v4	202	91.56 (Very High)
P30531	SLC6A1	P30531-F1-model_v4	599	87.88 (High)
P48066	SLC6A11	P48066-F1-model_v4	632	86.54 (High)
P48065	SLC6A12	P48065-F1-model_v4	614	87.34 (High)
Q9NSD5	SLC6A13	Q9NSD5-F1-model_v4	602	88.36 (High)
Q9UN76	SLC6A14	Q9UN76-F1-model_v4	642	89.82 (High)
Q9H2J7	SLC6A15	Q9H2J7-F1-model_v4	730	80.33 (High)
Q9GZN6	SLC6A16	Q9GZN6-F1-model_v4	736	79.76 (High)
Q9H1V8	SLC6A17	Q9H1V8-F1-model_v4	727	81.17 (High)
Q96N87	SLC6A18	Q96N87-F1-model_v4	628	90.46 (Very High)
Q695T7	SLC6A19	Q695T7-F1-model_v4	634	89.84 (High)
P23975	SLC6A2	P23975-F1-model_v4	617	87.21 (High)
Q9NP91	SLC6A20	Q9NP91-F1-model_v4	592	93.08 (Very High)
Q01959	SLC6A3	Q01959-F1-model_v4	620	86.55 (High)
P31645	SLC6A4	P31645-F1-model_v4	630	84.74 (High)
Q9Y345	SLC6A5	Q9Y345-F1-model_v4	797	73.5 (High)
P31641	SLC6A6	P31641-F1-model_v4	620	87.27 (High)
Q99884	SLC6A7	Q99884-F1-model_v4	636	85.46 (High)
P48029	SLC6A8	P48029-F1-model_v4	635	85.18 (High)
P48067	SLC6A9	P48067-F1-model_v4	706	81.11 (High)
P30825	SLC7A1	P30825-F1-model_v4	629	81.77 (High)
Q9NS82	SLC7A10	Q9NS82-F1-model_v4	523	83.07 (High)
Q9UPY5	SLC7A11	Q9UPY5-F1-model_v4	501	86.46 (High)
Q8TCU3	SLC7A13	Q8TCU3-F1-model_v4	470	88.23 (High)
Q8TBB6	SLC7A14	Q8TBB6-F1-model_v4	771	73.12 (High)
P52569	SLC7A2	P52569-F1-model_v4	658	81.04 (High)
Q8WY07	SLC7A3	Q8WY07-F1-model_v4	619	84.11 (High)
O43246	SLC7A4	O43246-F1-model_v4	635	80.43 (High)
Q01650	SLC7A5	Q01650-F1-model_v4	507	85.69 (High)
Q92536	SLC7A6	Q92536-F1-model_v4	515	82.89 (High)
Q9UM01	SLC7A7	Q9UM01-F1-model_v4	511	83.42 (High)
Q9UHI5	SLC7A8	Q9UHI5-F1-model_v4	535	81.51 (High)
P82251	SLC7A9	P82251-F1-model_v4	487	86.08 (High)
P32418	SLC8A1	P32418-F1-model_v4	973	75.23 (High)
Q9UPR5	SLC8A2	Q9UPR5-F1-model_v4	921	76.34 (High)
P57103	SLC8A3	P57103-F1-model_v4	927	76.4 (High)
Q6J4K2	SLC8B1	Q6J4K2-F1-model_v4	584	80.36 (High)
P19634	SLC9A1	7DSX	815	—
Q9UBY0	SLC9A2	Q9UBY0-F1-model_v4	812	66.19 (Low)
P48764	SLC9A3	7X2U	834	—
Q6AI14	SLC9A4	Q6AI14-F1-model_v4	798	66.65 (Low)
Q14940	SLC9A5	Q14940-F1-model_v4	896	62.93 (Low)
Q92581	SLC9A6	Q92581-F1-model_v4	669	70.34 (High)

Q96T83	SLC9A7	Q96T83-F1-model_v4	725	67.89 (Low)
Q9Y2E8	SLC9A8	Q9Y2E8-F1-model_v4	581	76.18 (High)
Q8IVB4	SLC9A9	Q8IVB4-F1-model_v4	645	73.36 (High)
Q4ZJI4	SLC9B1	Q4ZJI4-F1-model_v4	515	79.12 (High)
Q86UD5	SLC9B2	Q86UD5-F1-model_v4	537	78.93 (High)
Q4G0N8	SLC9C1	Q4G0N8-F1-model_v4	1177	74.33 (High)
Q5TAH2	SLC9C2	Q5TAH2-F1-model_v4	1124	77.57 (High)
P46721	SLCO1A2	P46721-F1-model_v4	670	80.86 (High)
Q9Y6L6	SLCO1B1	Q9Y6L6-F1-model_v4	691	80.68 (High)
Q9NPD5	SLCO1B3	Q9NPD5-F1-model_v4	702	79.32 (High)
G3V0H7	SLCO1B7	G3V0H7-F1-model_v4	640	82.39 (High)
Q9NYB5	SLCO1C1	Q9NYB5-F1-model_v4	712	78.22 (High)
Q92959	SLCO2A1	Q92959-F1-model_v4	643	81.65 (High)
O94956	SLCO2B1	O94956-F1-model_v4	709	78.54 (High)
Q9UIG8	SLCO3A1	Q9UIG8-F1-model_v4	710	75.65 (High)
Q96BD0	SLCO4A1	Q96BD0-F1-model_v4	722	77.85 (High)
Q6ZQN7	SLCO4C1	Q6ZQN7-F1-model_v4	724	78.35 (High)
Q9H2Y9	SLCO5A1	Q9H2Y9-F1-model_v4	848	70.18 (High)
Q86UG4	SLCO6A1	Q86UG4-F1-model_v4	719	76.83 (High)
Q9H2V7	SPNS1	Q9H2V7-F1-model_v4	528	84.21 (High)
Q8IVW8	SPNS2	Q8IVW8-F1-model_v4	549	81.86 (High)
Q6ZMD2	SPNS3	Q6ZMD2-F1-model_v4	512	84.41 (High)
Q9BX79	STRA6	Q9BX79-F1-model_v4	667	77.65 (High)
Q7L0J3	SV2A	Q7L0J3-F1-model_v4	742	77.22 (High)
Q7L1I2	SV2B	Q7L1I2-F1-model_v4	683	82.57 (High)
Q496J9	SV2C	Q496J9-F1-model_v4	727	78.45 (High)
Q8N4V2	SVOP	Q8N4V2-F1-model_v4	548	78.23 (High)
Q8N434	SVOPL	Q8N434-F1-model_v4	492	83.59 (High)
Q6UWJ1	TMCO3	Q6UWJ1-F1-model_v4	677	77.18 (High)
Q8NE00	TMEM104	Q8NE00-F1-model_v4	496	80.58 (High)
Q8TC26	TMEM163	Q8TC26-F1-model_v4	289	76.21 (High)
Q9HC07	TMEM165	Q9HC07-F1-model_v4	324	75.93 (High)
Q6ZMB5	TMEM184A	Q6ZMB5-F1-model_v4	413	71.46 (High)
Q9Y519	TMEM184B	Q9Y519-F1-model_v4	407	72.59 (High)
Q9NVA4	TMEM184C	Q9NVA4-F1-model_v4	438	72.04 (High)
Q24JQ0	TMEM241	Q24JQ0-F1-model_v4	296	94.31 (Very High)
Q13454	TUSC3	Q13454-F1-model_v4	348	84.45 (High)
P25874	UCP1	P25874-F1-model_v4	307	72.13 (High)
P55851	UCP2	P55851-F1-model_v4	309	60.03 (Low)
P55916	UCP3	P55916-F1-model_v4	312	61.83 (Low)
Q9UBH6	XPR1	Q9UBH6-F1-model_v4	696	83.37 (High)

Table S3. Docking data summary

Due to the massive data, Table S3 is presented in the excel file named “Docking data summary”

Table S4. Interaction frequency

Due to the massive data, Table S4 is presented in the excel file named “interaction_frequency”

Table S5. Prolif hydrophobic

Due to the massive data, Table S5 is presented in the excel file named “prolif_hydrophobic”
